# Phytochemical Diversity, Extraction Strategies, Pharmacological Activities of *Larix sibirica*: A Comprehensive Review

**DOI:** 10.3390/molecules31203690

**Published:** 2026-10-15

**Authors:** Gulden Kurmangaliyeva, Gulbaram Ustenova, Mehmet Öztürk, Galiya Sayakova, Assyl Boshkayeva, Akerke Amirkhanova, Moldir Koilybayeva, Moldir Zhandabayeva, Arailym Abilbayeva, Aigerim Kantureyeva

**Affiliations:** 1 School of Pharmacy, S.D. Asfendiyarov Kazakh National Medical University, Tole Bi 94, Almaty 050012, Kazakhstan; ustenova.g@kaznmu.kz (G.U.); amirhanova.a@kaznmu.kz (A.A.); zhandabaeva.m@kaznmu.kz (M.Z.); kantureeva.a@kaznmu.kz (A.K.); 2 Department of Chemistry, Mugla Sıtkı Koçman University, Kotekli, Mugla 48187, Turkey; mehmetsadettin@gmail.com; 3 Department of Pharmacognosy with Botany Course, S.D. Asfendiyarov Kazakh National Medical University, Tole Bi 94, Almaty 050012, Kazakhstan; sayakova.g@kaznmu.kz; 4 Department of Pharmaceutical and Toxicological Chemistry, S.D. Asfendiyarov Kazakh National Medical University, Tole Bi 94, Almaty 050012, Kazakhstan; boshkaeva.a@kaznmu.kz; 5 Department of Chemistry, Faculty of Science and Technology, Lancaster University, Lancaster LA1 4YB, UK; 6 General Immunology Department, S.D. Asfendiyarov Kazakh National Medical University, Tole Bi 94, Almaty 050012, Kazakhstan; abilbaeva.a@kaznmu.kz

**Keywords:** *Larix sibirica*, phytochemical composition, phenolic compounds, terpenoids, arabinogalactan, pharmacological activities, drug-delivery systems

## Abstract

*Larix sibirica* is a conifer and a renewable source of chemically diverse constituents with pharmacological uses. This review examines its phytochemical composition, extraction strategies, and reported pharmacological activities, including preliminary studies of arabinogalactan-based drug carriers and sulfated lignins. The compiled database comprised 354 records representing 232 unique compounds reported in 25 publications, distributed among 16 phytochemical groups and 12 plant-source categories. Phenolics, terpenoids, lipids, arabinogalactan and lignans were concentrated unevenly across bark, needles, oleoresin, wood, knotwood and heartwood resin. Extraction remained dominated by conventional solvent partitioning, Soxhlet extraction, steam distillation and chromatography; aqueous ethanol enabled simultaneous recovery of arabinogalactan and taxifolin, but direct comparisons with intensified technologies were lacking. Antioxidant activity was supported mainly by chemical assays, whereas antiviral, antibacterial, neuritogenic, anti-inflammatory and anti-allergic effects relied predominantly on cellular or limited animal models. Arabinogalactan showed immunomodulatory and intestinal barrier effects and was investigated as a preclinical drug carrier, while sulfated lignins showed concentration-dependent effects in in vitro blood-compatibility assays. Nevertheless, heterogeneous preparations, inconsistent measurement bases and limited dose–response, pharmacokinetic and toxicological data restrict cross-study comparison and clinical interpretation. Future work should prioritize standardized sampling and extracts, validated quantitative LC-MS/MS and GC-MS methods, untargeted metabolomics, bioactivity-guided fractionation, controlled in vivo studies and systematic safety assessment. These advances are required to convert descriptive phytochemistry into reproducible evidence for therapeutic and industrial development.

## 1. Introduction

*Larix sibirica* Ledeb. (Siberian larch) is a deciduous coniferous tree of the family Pinaceae and genus Larix. It is naturally distributed across northern Eurasia, including Russia, Kazakhstan, Mongolia, and northwestern China, where it is one of the dominant forest-forming species of the boreal zone [1,2,3,4]. The species commonly occurs in boreal and montane forests, forming both pure and mixed stands under strongly continental climatic conditions [1,4]. Botanically, *L. sibirica* is characterized by seasonally shed needles arranged on short shoots, thick bark, resin-containing tissues, and woody cones typical of larches [2,3]. It exhibits high ecological plasticity and is well adapted to cold continental environments. However, its ecological niche differs from that of the closely related *L. gmelinii*, which is better adapted to continuous permafrost, whereas *L. sibirica* is generally more warmth-demanding and less tolerant of prolonged waterlogged conditions [4,5]. Its deciduous growth habit, annual needle renewal, and adaptive anatomical and physiological characteristics contribute to its persistence under cold, drought, and other environmental stresses, making it an important component of boreal forest ecosystems [2,4].

*L. sibirica* represents an economically important forest resource that supports the timber, pulp and paper, and wood-processing industries. Owing to its high-density, durable, and decay-resistant wood, it has long been utilized for structural timber, construction materials, furniture, and engineered wood products [1,4]. The species also plays an important role in sustainable forestry and reforestation programs because of its adaptability to harsh climatic conditions and its capacity to establish productive forest stands [1]. In addition to its conventional utilization as timber, *L. sibirica* has attracted increasing industrial interest as a renewable feedstock for integrated forest biorefineries. Unlike most commercial softwoods, larch wood contains exceptionally high amounts of the water-soluble hemicellulose arabinogalactan [6], enabling efficient extraction under relatively mild hydrothermal conditions and reducing energy requirements during processing [7,8]. The recovered hemicellulose fraction can subsequently serve as a feedstock for the production of xylitol, barrier films, ethanol, jet and diesel fuels, gasoline, furfural, formic and acetic acids, and lactic acid [9,10,11]. Consequently, *L. sibirica* biomass has emerged as a promising renewable feedstock for forest biorefineries, facilitating the production of high-value biochemicals and biomaterials while maximizing the utilization of lignocellulosic resources without competing with food or feed production [12,13].

Beyond its ecological and economic importance, *L. sibirica* has gained considerable recognition as a medicinally valuable species owing to its remarkable phytochemical diversity and the abundance of pharmacologically relevant natural products. Among the species of the genus *Larix*, *L. sibirica* has emerged as one of the most extensively investigated sources of flavonoids, diterpenoids, polysaccharides, lignans, and volatile terpenoids. One of the earliest phytochemical investigations dedicated specifically to *L. sibirica* was reported in 1964 with the characterization of neutral diterpenoids from Siberian larch oleoresin, providing one of the earliest chemical descriptions of this species and laying the foundation for subsequent phytochemical studies [14]. Since then, characteristic diterpenoids, including (+)-larixol and larixyl acetate, have been identified in *L. sibirica* and other *Larix* species, becoming important subjects of phytochemical, synthetic, and biological investigations because of their distinctive chemical structures and pharmaceutical potential [14]. More recently, research has increasingly focused on taxifolin and arabinogalactan, two of the most abundant and extensively investigated constituents of *L. sibirica*, reflecting growing interest in their therapeutic and industrial applications [15,16]. In addition to these major compounds, needles are rich in volatile terpenoids dominated by δ-3-carene [17], α-pinene, β-pinene, myrcene, β-phellandrene, limonene, and bornyl acetate [18], whereas the bark, wood, resin, and oleoresin constitute valuable sources of flavonoids, diterpenoids, lignans, and polysaccharides [14]. Collectively, these findings demonstrate that virtually every anatomical part of *L. sibirica* represents a valuable source of chemically diverse natural products with considerable pharmaceutical potential.

The medicinal importance of *L. sibirica* is further supported by its long-standing use in traditional medicine across northern Eurasia. Ethnopharmacological applications of *L. sibirica* are deeply rooted in the traditional medicine systems of Siberian indigenous peoples, including Evenki, Yakut, and Tuvan communities. Bark decoctions have been used to treat gastrointestinal disorders, particularly diarrhea and dysentery, applications consistent with the astringent properties of bark tannins and the antimicrobial activities of phenolic compounds [19,20]. Resin, known locally as “serka,” has traditionally been chewed to relieve toothache, prevent dental caries, and alleviate inflammatory conditions of the oral cavity and throat, practices supported by modern evidence demonstrating antibacterial and anti-inflammatory activities of resin-derived diterpenoids and phenolic constituents [20,21]. Needle infusions have also been consumed as a source of vitamin C and used for respiratory infections, reflecting the abundance of ascorbic acid and bioactive terpenoids in fresh needles. Poultices prepared from the inner bark have been applied to wounds and burns, potentially benefiting from the wound-healing and antimicrobial activities of bark flavonoids and lignans [21].

Despite its long-standing traditional use and growing scientific interest, evidence concerning *L. sibirica* remains fragmented across phytochemical, pharmacological, and technological studies. This review therefore provides an integrated assessment of the phytochemical diversity and tissue distribution of its reported constituents, the extraction and analytical approaches used for their characterization, and the evidence for their pharmacological activities, including preliminary studies of drug-carrier systems and material compatibility. Particular attention is given to the distinction between qualitative identification and quantitative characterization, variation among plant sources, and the methodological limitations that constrain comparisons across studies. By integrating the available evidence, this review identifies the most consistently supported activities and applications while defining the analytical, toxicological, and translational priorities required for the future development of *L. sibirica*-derived compounds and materials.

## 2. Methods

This comprehensive review was conducted using a structured narrative approach to integrate phytochemical, extraction and pharmacological evidence concerning *L. sibirica* Ledeb. The review design followed established recommendations for transparent literature synthesis and narrative-review reporting [22,23], while documentation of the search strategy was informed by PRISMA-S guidance [24].

**Figure 1:**
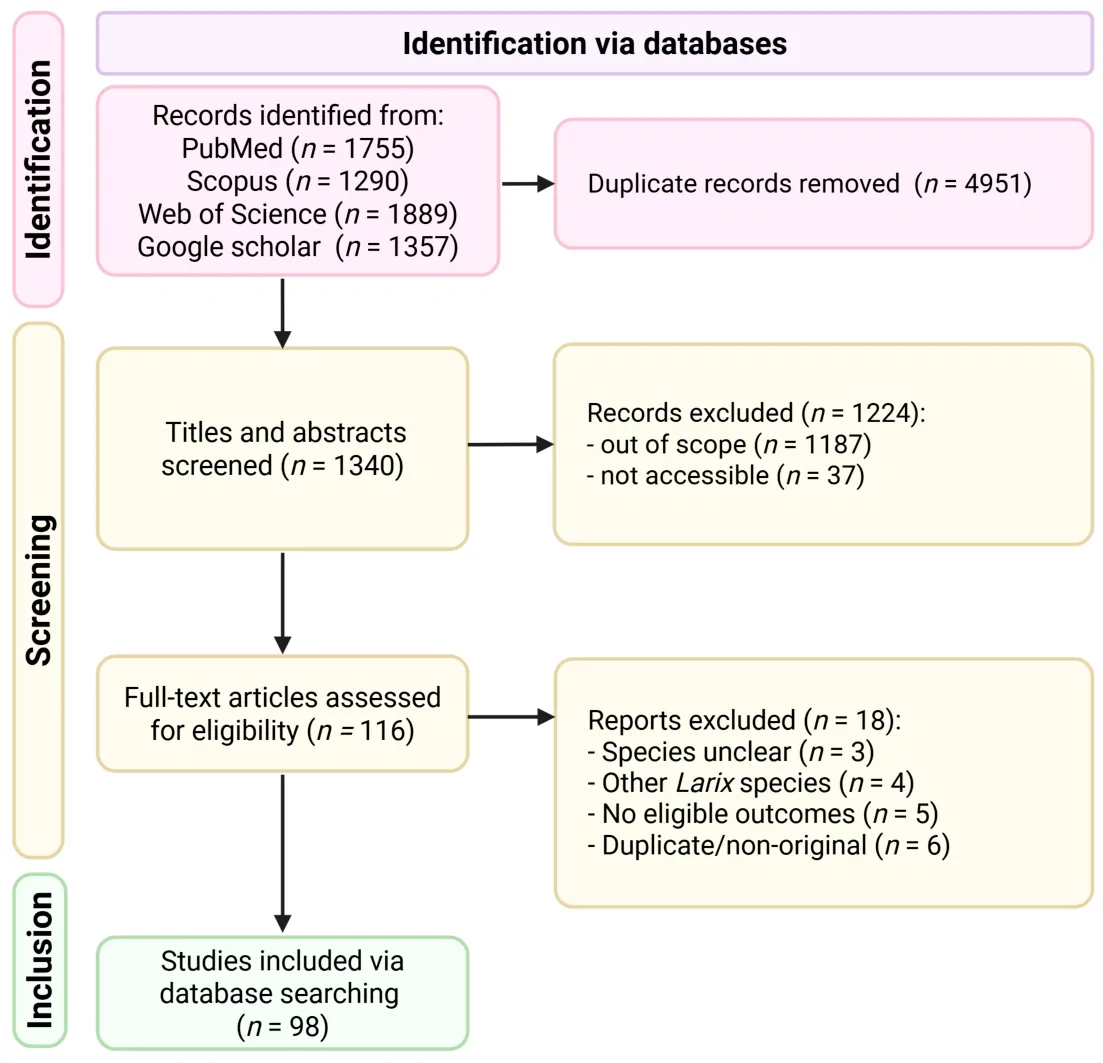
Flow diagram of literature identification, screening, eligibility assessment and study inclusion. Records were retrieved from PubMed, Scopus, Web of Science and Google Scholar, followed by duplicate removal, joint title-and-abstract screening, and full-text assessment.

Scopus, Web of Science, PubMed and Google Scholar were searched between 10 April and 15 July 2026, without restricting publication year (Figure 1). The principal search strategy combined the species name with terms related to chemical composition and biological activity: (“*Larix sibirica*” OR “Siberian larch”) AND (phytochemical* OR “chemical composition” OR metabolite* OR compound* OR extract* OR flavonoid* OR phenolic* OR terpen* OR lignan* OR lipid* OR sterol* OR arabinogalactan OR pharmacolog* OR biolog*). The search syntax was adapted to the requirements of each database. Additional searches combined the species name with “extraction”, “isolation”, “antioxidant”, “antiviral”, “antibacterial”, “anti-inflammatory”, “anti-allergic”, “neuroprotective”, “immunomodulatory”, “anticancer”, “hemocompatibility” and related terms. Russian-language equivalents and transliterated species names were used to identify regional literature. Reference lists of eligible articles and relevant reviews were screened to recover older studies that were not consistently indexed in electronic databases.

Original studies were eligible when they explicitly identified *L. sibirica* as the investigated species and reported at least one of the following: (i) isolation, identification or quantification of a chemical constituent; (ii) extraction, fractionation or analytical characterization of a species-derived preparation; or (iii) evaluation of the biological, pharmacological or biomedical properties of an extract, fraction, isolated compound or chemically modified material derived from *L. sibirica*. Studies involving several *Larix* or conifer species were included only when the results for *L. sibirica* could be extracted separately. No language restrictions were applied. Relevant publications in English, Russian and Chinese were identified and considered for inclusion.

Records were excluded when the plant material was described only as “larch” without reliable species identification, when the reported material originated exclusively from another *Larix* species, or when compound occurrence could not be traced to primary experimental evidence. Duplicate reports were consolidated, whereas distinct experiments reported in the same publication were retained when they concerned different plant sources or analytical observations. Reviews and studies of compounds obtained from other species were used only to provide mechanistic or comparative context and were not incorporated into the *L. sibirica* phytochemical dataset.

Eligible phytochemical studies were entered into a record-level database presented in Table S1. For each observation, the compound name, original plant source, standardized plant-source category, phytochemical group, chemical class, reference, publication year, quantitative value, unit, measurement basis and quantification status were recorded. Compound names and obvious orthographic or synonymous variants were standardized without altering the chemical identity reported by the original authors. Stereoisomers were treated as distinct compounds when explicitly differentiated; unresolved designations, including combined cis/trans forms, were retained as reported.

A record was defined as the occurrence of a compound in a specific plant source and publication. Consequently, the same compound reported in different tissues or independent studies generated multiple records. By contrast, overall chemical diversity was calculated after deduplication of standardized compound names. A compound–source occurrence represented each unique combination of compound and standardized plant-source category. Numerical results were retained using their original units and measurement bases because the reported denominators, including dry tissue, essential oil, total extract, resinous compounds, isolated fractions, extraction yield and purity, were not directly interchangeable. Observations reported as “identified only” or “trace” were retained as qualitative evidence but were not considered quantitative measurements.

The evidence was synthesized descriptively because marked heterogeneity in plant material, extraction procedures, analytical platforms, experimental models, doses and outcome definitions precluded a meaningful meta-analysis. Temporal analysis was based on the number of eligible publications and unique compounds reported in each publication year. Chemical distribution was examined by calculating the number of unique compounds assigned to each phytochemical group and plant-source category; within-source percentages were calculated relative to the total number of unique compounds reported for the corresponding source. Descriptive calculations and graphical analyses were performed using R (R Foundation for Statistical Computing, Vienna, Austria) through RStudio (version 2025.05.0+496; Posit Software, PBC, Boston, MA, USA).

Critical interpretation considered the botanical origin and preparation of the test material, extraction and fractionation procedures, analytical support for compound identification, measurement basis used for quantitative data, experimental model, dose and exposure conditions, comparator selection, statistical reporting and directness of the evidence. Particular caution was applied when biological mechanisms were inferred solely from pathway-associated markers, when compounds were evaluated at a single concentration, or when findings from other *Larix* species were extrapolated to *L. sibirica*. Accordingly, the review distinguishes direct species-specific evidence from supporting evidence derived from related compounds, preparations or experimental systems.

## 3. Phytochemical Composition of *L. sibirica*

### 3.1. Overview of Phytochemical Investigations

Phytochemical investigations of *Larix sibirica* have been conducted for nearly six decades and demonstrate a gradual transition from the isolation of individual metabolites to comprehensive phytochemical characterization (Table 1). One of the earliest published phytochemical studies, reported in 1967, identified the major bark flavonols, while another study published in the same year elucidated the structure of the diterpene diol 13-epitorulosol from oleoresin [25,26]. These investigations provided the foundation for subsequent phytochemical research. During the late 1960s and 1970s, research expanded to the characterization of oleoresin terpenoids, bark flavonoids, needle flavonoid glycosides, and wood polysaccharides. Numerous monoterpenes, sesquiterpenes, flavonoids, catechins, and arabinogalactan were identified using chromatographic and spectroscopic techniques [27,28,29,30,31,32,33]. During the 1980s and 1990s, investigations focused on volatile compounds from female flowers, prostaglandins from cambial tissues, and lipid constituents of needles and woody verdure [34,35,36]. Since 2003, phytochemical research has become increasingly comprehensive. Advanced analytical techniques enabled detailed characterization of bark polyphenols, heartwood resin constituents, knotwood lignans, branch bark terpenoids, lignin, sterols, triterpenoids, fatty acids, and essential oils from different plant tissues [37,38,39,40,41,42,43,44,45,46,47,48,49,50,51,52,53,54,55,56]. More recent studies have also investigated geographical variation, environmental responses, extraction optimization, and phytochemical profiles of in vitro cell cultures, further expanding the known chemical diversity of *L. sibirica*.

Bark has been the most extensively investigated plant material and remains the principal source of identified phytochemicals. Numerous studies have characterized bark flavonoids, phenolic acids, proanthocyanidins, condensed tannins, and other polyphenols [25,28,29,37,38,39]. Oleoresin represents the second most intensively studied source. Early investigations focused on monoterpenes and sesquiterpenes, whereas later studies expanded to diterpenoids and resin acids [26,27,31,40]. Needles have also received substantial attention, particularly for flavonoids, fatty acids, resin acids, wax constituents, triterpenoids, phytosterols, and stress-related metabolites [30,45,46,47,48,50,54,56]. Additional investigations have examined female flowers, woody verdure, twigs, branch bark, knotwood, heartwood resin, wood, cambial tissues, buds, crowns, and embryogenic cell cultures, although these plant parts have been studied much less frequently [32,33,34,35,36,40,41,42,43,44,49,51,52,53,55]. In contrast, several tissues remain poorly explored or have been investigated only once. These include the cambial zone and buds, female flowers, spring needles, branch bark, woody greens, embryogenic cell cultures, knotwood, and wood lignin. Roots, seeds, cones, pollen, and reproductive tissues other than female flowers have not been phytochemically characterized to date, indicating important directions for future investigations.

Phytochemical methodologies applied to *L. sibirica* have undergone substantial development over the past six decades. Among the earliest studies, sample preparation was relatively simple and mainly involved solvent extraction followed by repeated purification using paper or column chromatography [25,26,27,28,29,30,31]. These approaches were suitable for isolating abundant flavonoids, terpenoids, and catechins but provided limited chemical coverage. During the 1980s and 1990s, extraction protocols became more specialized. Steam distillation, headspace sampling, gel filtration, lipid extraction, and derivatization were introduced according to the physicochemical properties of the target metabolites [32,33,34,35,36]. Since the early 2000s, sample preparation has become increasingly selective and application-oriented. Sequential solvent extraction, Soxhlet extraction, flash chromatography, solid-phase fractionation, supercritical fluid extraction, mechanical activation, and cell culture techniques have enabled efficient isolation of structurally diverse metabolites from different plant tissues [37,38,39,40,41,42,43,44,45,46,47,48,49,50,51,52,53,54,55,56]. Nevertheless, most protocols remain targeted toward specific phytochemical groups. Universal extraction strategies capable of recovering the full metabolite spectrum have rarely been applied.

Analytical techniques have evolved in parallel with sample preparation and have markedly improved the phytochemical characterization of *L. sibirica*. Early investigations relied primarily on paper chromatography together with UV, IR, and one-dimensional NMR spectroscopy, which allowed identification of major constituents but offered limited structural resolution [25,26,27,28,29,30,31]. The introduction of gas chromatography substantially improved the analysis of volatile terpenoids and essential oils [27,31,32]. Later studies combined chromatographic separation with HPLC, GC–MS, multidimensional NMR, HRMS, and supercritical fluid chromatography, enabling the identification of complex polyphenols, lignans, diterpenoids, sterols, and lipid-derived metabolites with much greater confidence [37,38,39,40,41,42,43,44,45,46,47,48,49,50,51,52,53,54,55,56]. Despite these advances, the current literature remains dominated by targeted phytochemical analyses. High-throughput metabolomics, quantitative LC–MS/MS profiling, and integrated multi-omics approaches have been applied only rarely. As a result, the global phytochemical diversity of *L. sibirica* is likely to remain incompletely characterized.

### 3.2. Diversity and Distribution of Reported Phytochemicals

**Figure 2:**
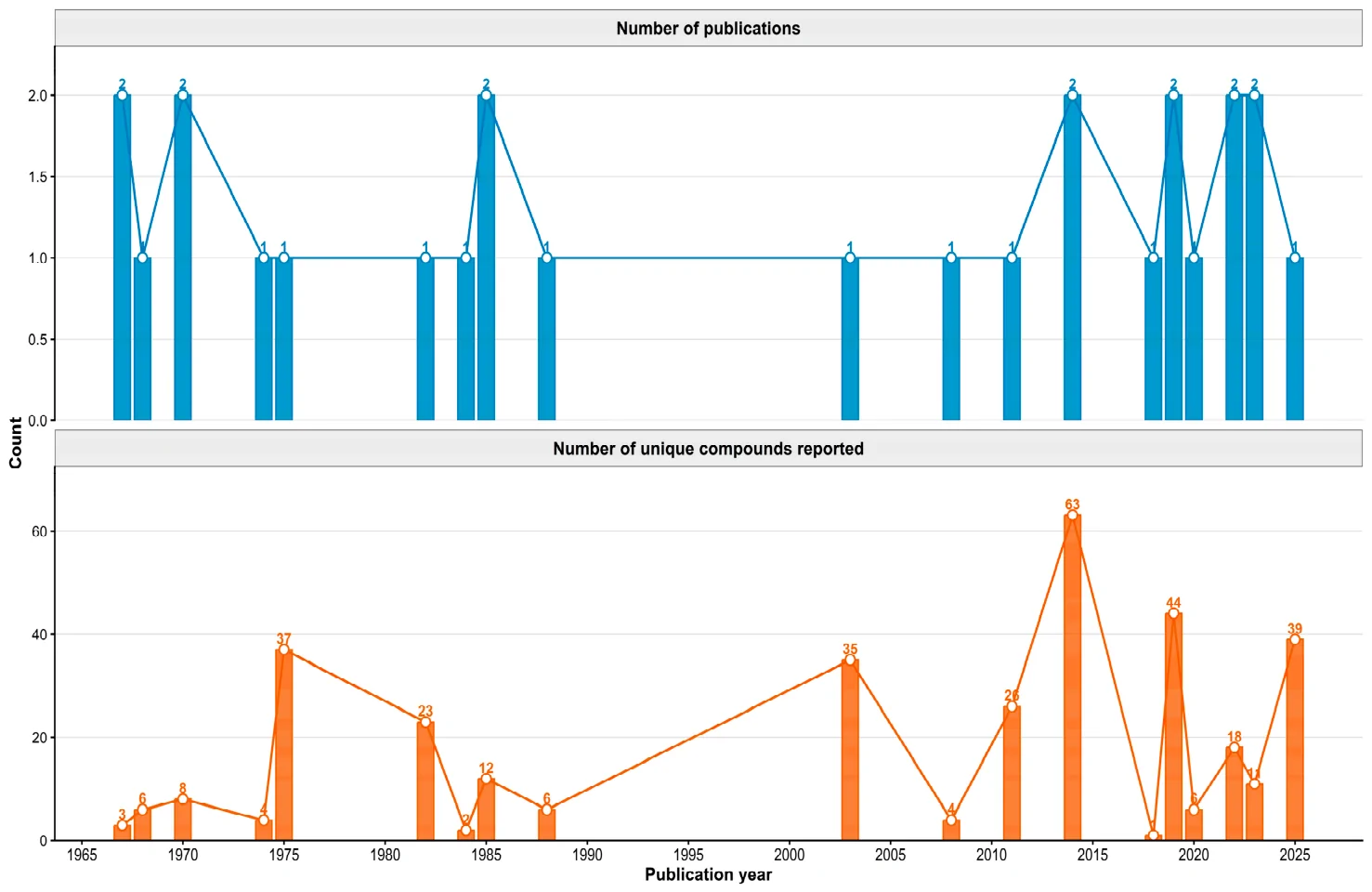
Temporal trends in phytochemical investigations of *L. sibirica*.

Phytochemical investigations of *Larix sibirica* have been conducted for nearly six decades, beginning with studies of bark flavonols and oleoresin diterpenoids in 1967 and subsequently expanding to a broader range of plant materials and chemical constituents (Table 1). Despite this long research period, publication activity remained intermittent: studies were reported in only 19 individual years between 1967 and 2025, with no more than two publications appearing in any single year (Figure 2). The number of unique compounds reported annually varied much more substantially than the number of publications, with pronounced increases in 1975 (37 compounds), 2003 (35 compounds), 2014 (63 compounds), 2019 (44 compounds), and 2025 (39 compounds) [31,37,41,42,46,47,55]. The largest expansion occurred in 2014, when two studies collectively reported 63 unique compounds, whereas several other publication years contributed fewer than ten compounds. Thus, the growth of the documented phytochemical diversity of *L. sibirica* was driven primarily by a limited number of compound-rich investigations rather than by a consistent increase in annual publication output.

Across the 25 phytochemical studies, 354 compound records were documented, corresponding to 232 unique compounds identified in 12 plant-source categories (Table 2 and Table S1). These compounds covered 16 phytochemical groups: terpenoids, diterpenoids, triterpenoids, flavonoids, phenolics, phenolic acids, polyphenols, lignans, oligolignans, phenylpropanoids/benzenoids, fatty acids, lipids, phytosterols, polysaccharides, long-chain alcohols, and ketones. Terpenoids constituted the largest group, with 130 unique compounds (55.8% of the total), followed by flavonoids with 33 (14.2%), fatty acids with 21 (9.0%), phenolics with 11 (4.7%), and triterpenoids with nine (3.9%). The remaining 29 compounds (12.4%) were distributed among 11 less frequently reported groups. The 12 investigated plant sources comprised bark, branch bark, needles, oleoresin, heartwood resin, woody greens, woody verdure, wood, knotwood, female flowers, the cambial zone and buds, and cell cultures (Table 2 and Table S1).

**Figure 3:**
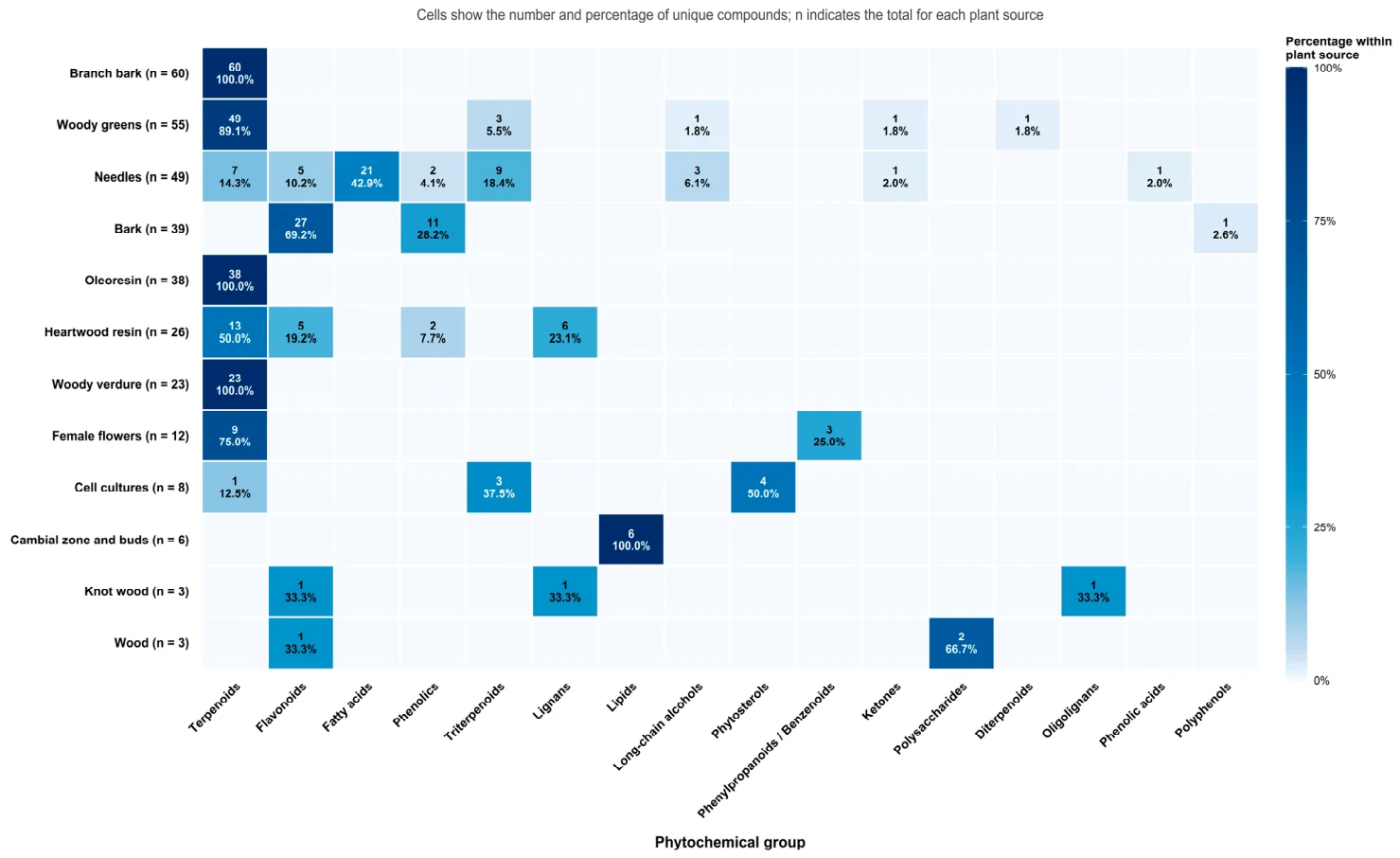
Distribution of reported phytochemicals by chemical group and plant source.

The distribution of phytochemical groups varied markedly among the investigated plant sources (Figure 3). Terpenoids accounted for all compounds reported from branch bark, oleoresin, and woody verdure and represented 89.1% of the compounds detected in woody greens. Female flowers were also dominated by terpenoids (75.0%), with phenylpropanoids/benzenoids accounting for the remaining 25.0%. In contrast, bark predominantly contained flavonoids (69.2%) and phenolics (28.2%), while heartwood resin showed a more heterogeneous composition comprising terpenoids (50.0%), lignans (23.1%), flavonoids (19.2%), and phenolics (7.7%). Needles exhibited the broadest chemical distribution, encompassing eight phytochemical groups and being particularly enriched in fatty acids (42.9%), triterpenoids (18.4%), terpenoids (14.3%), and flavonoids (10.2%). Distinct profiles were also observed in the less extensively represented sources: lipids accounted for all compounds reported from the cambial zone and buds, phytosterols and triterpenoids predominated in cell cultures, and polysaccharides constituted two-thirds of the compounds identified in wood (Figure 3).

The documented phytochemical diversity was distributed unevenly among plant sources (Figure 3). Branch bark contained the largest number of reported unique compounds (n = 60), followed by woody greens (n = 55), needles (n = 49), bark (n = 39), and oleoresin (n = 38). Intermediate numbers were recorded for heartwood resin (n = 26) and woody verdure (n = 23), whereas considerably fewer compounds were reported from female flowers (n = 12), cell cultures (n = 8), the cambial zone and buds (n = 6), knotwood (n = 3), and wood (n = 3). However, high numerical diversity did not necessarily correspond to broad chemical diversity: branch bark, oleoresin, and woody verdure contained numerous reported compounds but were represented exclusively by terpenoids, whereas needles contained fewer compounds than branch bark or woody greens but covered the greatest number of phytochemical groups. These patterns characterize the current distribution of published evidence and should not be interpreted as definitive differences in the inherent chemical richness of the respective plant tissues.

### 3.3. Phenolic Compounds

#### 3.3.1. Phenolic Acids and Their Derivatives

Fifteen phenolic acids and related derivatives were identified across four plant sources, producing 19 unique compound–source occurrences and 22 individual literature records (Table S2). Bark was the principal source, containing 11 compounds: 4-hydroxybenzoic, caffeic, ferulic, protocatechuic, syringic, vanillic, *p*-coumaric, and *p*-hydroxybenzoic acids, as well as eicosanyl *p*-coumarate, eicosanyl ferulate, and docosanyl ferulate [37,39]. Three volatile derivatives, including ethyl benzoate, methyl salicylate, and ethyl salicylate, were reported exclusively in female flowers [34]. Needles contained cinnamic, vanillic, and *p*-hydroxybenzoic acids [46], whereas heartwood resin contained vanillic and ferulic acids [39,40]. Overall, the reported phenolic acid profile was concentrated primarily in bark, while other plant sources contained fewer and less diverse compounds.

Most compounds were specific to a single plant source, while only three of the 15 compounds were reported from more than one source. Vanillic acid showed the broadest distribution, occurring in bark, needles, and heartwood resin [37,39,40,46]. *p*-Hydroxybenzoic acid was shared between bark and needles [37,46], whereas ferulic acid was reported from bark and heartwood resin [37,39,40]. The remaining 12 compounds were confined to one source in the available literature. These included several bark-specific phenolic acids and long-chain phenolic esters [37,39], three volatile benzoate and salicylate derivatives restricted to female flowers [34], and cinnamic acid detected only in needles [46]. The limited overlap indicates pronounced source-associated differentiation in the reported profiles; however, it may also reflect the selective examination of particular tissues and compound classes rather than complete chemical separation among plant parts.

Quantitative information was available for only five of the 22 records. In needles, vanillic acid and *p*-hydroxybenzoic acid were reported at concentrations ranging from trace levels to 0.16% and 0.18% of dry weight, respectively [46]. In female flowers, ethyl benzoate, methyl salicylate, and ethyl salicylate each represented less than 1% of the total volatile fraction [34]. Cinnamic acid was detected only at trace levels in needles [46], while the remaining compounds, including most of those identified in bark and heartwood resin, were reported without quantitative concentrations [37,39,40]. Therefore, the available studies provide considerably stronger evidence for the chemical diversity and plant source distribution of these compounds than for their relative abundance. Differences in measurement basis also prevent direct quantitative comparisons among needles, female flowers, bark, and heartwood resin.

#### 3.3.2. Flavonoids and Flavonoid Glycosides

Twenty-six unique flavonoids and related compounds were reported from five plant sources, corresponding to 31 unique compound–source occurrences across 38 literature records from eight publications (Table S2). The identified compounds represented several structural classes, including four flavonols, eight flavonol glycosides, three flavanones, two dihydroflavonols, two *O*-methylated flavonols, two structurally distinctive spiroflavonoids, and one anthocyanidin. Bark contained the greatest diversity, with 16 compounds, followed by heartwood resin with five, needles with four, and knotwood and wood with one compound each [25,28,30,37,40,41,50,53]. This distribution indicates that the current knowledge of *L. sibirica* flavonoids is based predominantly on bark, whereas the flavonoid profiles of other tissues remain comparatively limited.

The bark profile included the flavonol aglycones kaempferol, quercetin, myricetin, and isorhamnetin, together with the glycosides avicularin, guaijaverin, quercitrin, and three quercetin arabinosides [25,28,37]. Bark also contained the dihydroflavonols taxifolin and aromadendrin, the flavanone naringenin, the anthocyanidin listvenidin, and the structurally unusual compounds larixinol and triflarixinol [28,37,41]. In contrast, the needle profile was restricted to laricitrin, syringetin, laricitrin 3-glucoside, and syringetin 3-rutinoside [30]. The exclusive occurrence of these methylated flavonols and their glycosides in needles distinguishes the reported needle profile from the more structurally diverse mixture of aglycones and glycosides identified in bark. However, this apparent tissue specificity should be interpreted cautiously because the two sources were not investigated with equivalent analytical coverage.

Most flavonoids were reported from only one plant source. Of the 22 unique compounds, 19 were source-specific within the available literature, while aromadendrin, naringenin, and taxifolin occurred in more than one source.Aromadendrin and naringenin were shared between bark and heartwood resin [28,37,40]. Taxifolin showed the broadest distribution and was identified in bark, heartwood resin, knotwood, and wood [28,37,40,41,50,53]. Heartwood resin additionally contained eriodictyol and pinocembrin, which were not reported from the other examined sources [40]. Consequently, taxifolin represents the only flavonoid documented across four structurally distinct plant materials, whereas the remaining profile consists largely of compounds reported from individual tissues.

Quantitative data were available for seven of the 22 unique flavonoids. In bark, kaempferol and quercetin were reported at 0.1% and 0.006% of dry bark, respectively [25,37]. In heartwood resin, taxifolin accounted for 12% and aromadendrin for 1.3% of total resinous compounds, while eriodictyol, naringenin, and pinocembrin were each reported at less than 0.5% [40]. Taxifolin was also reported at 2% of dry wood, with an extraction yield of 1.81%, and at an extraction yield of 6.3% from knotwood, where the isolated product reached 95% purity [51,53]. These values demonstrate the quantitative prominence of taxifolin in several woody sources, but they cannot be directly compared because they refer to different measurement bases, including dry material content, proportion of resinous compounds, extraction yield, and product purity. Furthermore, most bark and all needle flavonoids were identified without quantitative concentration data, limiting comparisons of flavonoid abundance among plant sources.

#### 3.3.3. Catechins, Proanthocyanidins, and Tannins

Twelve catechin-related compounds, proanthocyanidins, and tannins were reported from bark and needles, corresponding to 13 compound–source occurrences and 16 literature records from three publications (Table S2). The identified constituents included four flavan-3-ols, namely (+)-catechin, (−)-epicatechin, (−)-epiafzelechin, and (+)-gallocatechin; three flavan-3-ol glycosides, including catechin 7-*O*-β-glucopyranoside, catechin rhamnoside, and epicatechin 7-*O*-β-glucopyranoside; four dimeric procyanidins, B1, B2, B3, and B4; and condensed tannins reported as a collective compound group [29,37]. This profile demonstrates the presence of both monomeric flavan-3-ols and their glycosylated and oligomeric derivatives in *L. sibirica*.

Bark contained all 12 reported constituents and was therefore the principal source of this phytochemical group [29,37]. (+)-Catechin, (−)-epicatechin, and (−)-epiafzelechin were reported in more than one bark study, whereas (+)-gallocatechin, the three flavan-3-ol glycosides, procyanidins B1 to B4, and condensed tannins were each documented in a single study [29,37]. Needles contained only (+)-catechin, making this compound the sole constituent reported from both plant sources [29,37,45]. The remaining 11 constituents were restricted to bark in the available literature. This marked concentration in bark may indicate a tissue-associated enrichment of flavan-3-ol derivatives and condensed polyphenols, but it also reflects the limited investigation of these compounds in needles and the absence of comparable data for other plant tissues.

Quantitative characterization was extremely limited. Of the 16 literature records, only one included a numerical measurement: needle catechin showed a 3.0-fold to 4.8-fold relative increase in the investigated comparison [45]. However, this result represents a relative change rather than an absolute concentration and therefore does not establish the catechin content of the needles. Epicatechin was detected only at trace levels in bark, while the remaining catechins, glycosides, procyanidins, and condensed tannins were reported qualitatively without concentration values [29,37]. The available evidence therefore confirms structural diversity within this group but does not permit reliable comparisons of abundance among individual compounds or plant sources.

#### 3.3.4. Lignans and Oligolignans

Seven lignan-related compounds were reported from heartwood resin and knotwood, corresponding to eight compound–source occurrences and 12 literature records from three publications (Table S2). The identified compounds included six lignans, namely (+)-isolariciresinol, (−)-secoisolariciresinol, 3,4-divanillyltetrahydrofuran, conidendrin, lariciresinol, and pinoresinol, together with the oligolignan secoisolariciresinol-sesquilignan [40,41,53]. Heartwood resin contained six of the seven compounds, whereas knotwood contained two, indicating that the documented lignan diversity of *L. sibirica* is concentrated primarily in woody resinous tissues.

Heartwood resin contained (+)-isolariciresinol, (−)-secoisolariciresinol, 3,4-divanillyltetrahydrofuran, conidendrin, lariciresinol, and pinoresinol [40,41]. Knotwood contained (−)-secoisolariciresinol and secoisolariciresinol-sesquilignan [53]. Among the seven compounds, (−)-secoisolariciresinol was the only constituent reported from both heartwood resin and knotwood, while the other six were restricted to a single source in the available literature. (+)-Isolariciresinol and (−)-secoisolariciresinol were each confirmed by two independent studies of heartwood resin [40,41], whereas the other heartwood lignans were reported in only one study [40]. Thus, the apparent differences between heartwood resin and knotwood remain based on limited and uneven research coverage rather than comprehensive comparative profiling.

Quantitative data were available only for the two compounds isolated from knotwood. (−)-Secoisolariciresinol had an extraction yield of 23%, and the isolated product reached 91% purity, whereas secoisolariciresinol-sesquilignan had an extraction yield of 2.4% and a purity of 92% [53]. These measurements describe extraction performance and product purity rather than the native concentrations of the compounds in knotwood. No quantitative values were reported for the six lignans identified in heartwood resin [40,41]. Consequently, the available evidence supports the presence and structural diversity of lignans in *L. sibirica* but does not permit direct comparison of their natural abundance in heartwood resin and knotwood.

### 3.4. Terpenoids and Resin Constituents

#### 3.4.1. Monoterpenes

Thirty-one unique monoterpenes were reported from five plant sources, corresponding to 76 compound–source occurrences and 83 individual records from eight publications (Table S3). The reported profile comprised 17 monoterpene hydrocarbons, nine monoterpene alcohols, two oxygenated monoterpenes, one monoterpene ester, one monoterpene ketone, and one monoterpene phenol. Woody greens contained the greatest number of compounds (n = 22), including α-pinene, β-pinene, 3-carene, limonene, myrcene, β-phellandrene, and several oxygenated derivatives such as linalool, thymol, verbenone, and cymen-8-ols [49,55]. Branch bark contained 17 compounds and was characterized by 3-carene, terpinolene, γ-terpinene, β-pinene, myrcene, and several less widely distributed constituents, including sabinene, α-fenchene, α-thujene, and β-ocimene [42]. Fifteen compounds were reported from oleoresin, with 3-carene, α-pinene, β-pinene, β-phellandrene, myrcene, and limonene forming the principal hydrocarbon fraction [27,31]. Woody verdure contained 13 compounds, predominantly 3-carene, α-pinene, β-pinene, limonene, and β-phellandrene, together with the oxygenated monoterpenes borneol, camphor, and isoborneol [32]. Female flowers contained nine compounds, namely 3-carene, myrcene, β-phellandrene, α-pinene, β-pinene, limonene, terpinolene, borneol, and bornyl acetate [34,35]. Collectively, these findings reveal a shared monoterpene core across the volatile and resinous fractions of *L. sibirica*, accompanied by a smaller set of source-associated compounds.

Seven compounds showed particularly broad distributions. 3-Carene, limonene, myrcene, terpinolene, α-pinene, β-pinene, and β-phellandrene were each reported from all five plant sources [27,31,32,34,35,42,49]. In addition, camphene, borneol, and γ-terpinene occurred in four sources, whereas bornyl acetate, *p*-cymene, and α-phellandrene were found in three. By contrast, several compounds were reported from only one source. Branch bark contained dehydro-*p*-cymene, sabinene, α-fenchene, α-terpinene, α-thujene, and β-ocimene [42], while woody greens contained linalool, thymol, verbenone, *cis*-carveol, α-fenchol, *m*-cymen-8-ol, and *p*-cymen-8-ol [55]. Camphor and isoborneol were restricted to woody verdure [32], and thymol methyl ether was reported only from oleoresin [31]. Female flowers did not contain any source-specific monoterpenes in the available records, as all nine compounds reported from this source were also found in other tissues.

Despite broad qualitative overlap, the relative composition of the monoterpene fractions differed substantially among plant sources. 3-Carene was the most consistently prominent constituent, accounting for 49% and 22.3% of oleoresin, 53.11% of woody verdure essential oil, more than 20% of female flower volatiles, and 18.7% of branch bark essential oil [27,31,32,34,42]. Its reported proportion in woody greens varied from 1.5% to 48.7%, indicating considerable variability even within the same general plant material [49]. Oleoresin was additionally characterized by high proportions of α-pinene, β-pinene, β-phellandrene, myrcene, and limonene, although their reported values differed between studies [27,31]. For example, the two studies reported different proportions of α-pinene (5.8% and 30%), myrcene (2% and 12.4%), and limonene (3.7% and 10%), respectively; these values do not represent changes over time or responses to a treatment [27,31]. Woody verdure showed a more stable 3-carene-dominated profile, with α-pinene, β-pinene, limonene, and β-phellandrene as secondary constituents [32]. In branch bark, 3-carene remained predominant, followed by terpinolene, γ-terpinene, and β-pinene [42].

Quantitative values were available for 67 of the 83 records, providing substantially greater numerical coverage than for most nonvolatile phytochemical groups. Nevertheless, direct comparisons require caution because the reported percentages were calculated using different measurement bases, including total essential oil, total flower volatiles, oleoresin composition, and isolated monoterpene alcohol fractions. For example, α-terpineol represented more than 50% of the monoterpene alcohol fraction in oleoresin but only 0.24% of the total essential oil from woody greens [31,55]. These values describe different chemical fractions and therefore do not demonstrate a direct difference in tissue abundance. Moreover, the broad concentration ranges reported for 3-carene, α-pinene, and β-pinene in woody greens suggest substantial within-source variability [49]. Consequently, the available evidence identifies a shared core of widely distributed monoterpenes, but differences in plant material and measurement basis limit direct quantitative comparison of monoterpene profiles across studies.

#### 3.4.2. Sesquiterpenes

Fifty unique sesquiterpenes were reported from four plant sources, corresponding to 67 compound-source occurrences and 68 literature records from five publications (Table S3). These compounds comprised 41 sesquiterpene hydrocarbons and nine sesquiterpene alcohols. Branch bark contained 23 compounds: cadalene, caryophyllene, copaiene, cyclosativene, germacrene D, isocaryophyllene, longicyclene, longifolene, α-bisabolene, α-calacorene, α-curcumene, α-farnesene, α-himachalene, α-humulene, α-longipinene, α-muurolene, β-bisabolene, β-cedrene, β-elemene, β-farnesene, β-himachalene, β-selinene, and δ-cadinene [42]. Oleoresin contained 22 compounds: calamenene, caryophyllene, cyclosativene, longifolene, α-amorphene, α-cadinene, α-calacorene, α-copaene, α-humulene, α-muurolene, α-ylangene, β-cadinene, β-copaene, β-ylangene, γ-amorphene, γ-cadinene, γ-elemene, γ-muurolene, δ-cadinene, δ-elemene, ε-bulgarene, and ε-muurolene [31]. Woody greens contained 12 compounds: 10-hydroxy-*cis*-calamenene, caryophyllene, humulenol, longiborneol, longifolene, *trans*,*trans*-farnesol, α-bisabolol, α-cadinol, α-humulene, δ-cadinol, τ-cadinol, and τ-muurolol [49,55]. Finally, woody verdure contained ten compounds: bisabolene, caryophyllene, isolongifolene, longifolene, sibirene, α-humulene, α-longipinene, α-muurolene, α-ylangene, and δ-cadinol [32]. Thus, branch bark and oleoresin showed the greatest sesquiterpene diversity and were dominated by hydrocarbon constituents, whereas woody greens contained a smaller but more oxygenated profile composed largely of sesquiterpene alcohols.

Only three sesquiterpenes were reported from all four plant sources. Caryophyllene, longifolene, and α-humulene occurred in branch bark, oleoresin, woody greens, and woody verdure [31,32,42,49]. α-Muurolene was shared among branch bark, oleoresin, and woody verdure, whereas cyclosativene, α-calacorene, α-longipinene, α-ylangene, δ-cadinene, and δ-cadinol each occurred in two sources [31,32,42,55]. Nevertheless, most constituents showed a more restricted distribution. Fifteen compounds were reported only from branch bark, 14 only from oleoresin, eight only from woody greens, and three only from woody verdure. Notably, the source-specific profile of branch bark consisted primarily of hydrocarbon sesquiterpenes, whereas that of woody greens was dominated by sesquiterpene alcohols. This contrast suggests a shift from hydrocarbon-rich profiles in bark and resin to a greater representation of oxygenated sesquiterpenes in leafy and twig-containing material.

Quantitative profiles also differed markedly among the investigated sources. In branch bark essential oil, longifolene, α-humulene, and caryophyllene were the principal sesquiterpenes, accounting for 6.4%, 6.2%, and 5.1%, respectively [42]. α-Longipinene contributed 2.8%, while δ-cadinene accounted for 1.1%; the remaining quantified constituents were generally present at 0.5% or less [42]. In woody verdure, α-humulene and α-muurolene were reported as a combined peak of 2.55%, while caryophyllene represented 1.02% and individually resolved α-muurolene represented 0.83% of the essential oil [32]. By contrast, γ-elemene was described as a major component of the oleoresin hydrocarbon fraction at approximately 30%, although most other oleoresin sesquiterpenes were identified without individual concentrations [31]. Among the oxygenated constituents of woody greens, τ-muurolol was the most abundant at 5%, followed by α-cadinol and δ-cadinol at 0.5% each and τ-cadinol at 0.33% [55].

Numerical values were available for 37 of the 68 records, whereas the remaining compounds were reported qualitatively or at trace levels. However, the quantitative evidence is unevenly distributed among sources. Branch bark was characterized almost entirely by individual essential oil percentages [42], while the oleoresin profile was largely qualitative apart from the approximate abundance of γ-elemene [31]. Moreover, the unresolved α-humulene and α-muurolene peak in woody verdure prevents assignment of the reported 2.55% value to either compound individually [32]. Differences in measurement basis, chromatographic resolution, and quantitative coverage therefore limit direct comparisons among studies. Overall, the available evidence supports a shared sesquiterpene core across *L. sibirica* tissues, but the apparent source-specific patterns remain influenced by differences in analytical coverage and reporting.

#### 3.4.3. Diterpenes and Diterpene Alcohols

Forty unique diterpenes and related derivatives were reported from four plant sources, corresponding to 42 compound-source occurrences and 42 literature records from four publications (Table S3). The reported profile comprised 19 compounds classified as diterpenes, 11 diterpene alcohols, two nor-diterpene alcohols, two diterpene diols, two oxidized diterpenoids, and one compound each classified as an aromatic diterpene alcohol, diterpene acetate, diterpene aldehyde, and diterpene ester. Branch bark contained the greatest number of compounds (n = 19): 18-norabieta-4,8,11,13-tetraene, 18-norabieta-8,11,13-triene, 18-norisopimara-4(19),7,15(16)-triene, 18-norsandaracopimara-4(19),8,15(16)-triene, 19-norabieta-8,11,13-triene, 8(9)-isopimaradiene, abietadiene, cembrene, *cis*-biformene, *cis*/*trans*-18-norabieta-3,8,11,13-tetraenes, dehydroabietadiene, isocembrene, isopimaradiene, neoabietadiene, neocembrene, palustradiene, sandaracopimaradiene, *trans*-biformene, and β-springene [42]. This profile consisted almost entirely of hydrocarbon diterpenes and their nor-derivatives.

Woody greens contained 16 compounds: 13-epimanool, 15-hydroxymethyldehydroabietate, 18-nordehydroabietinol, 18-norisopimarinol, 7-oxo-methyldehydroabietate, abietinol, *cis*-abienol, dehydroabietinol, epitorulosol, epitorulosol acetate, geranyllinalool, isocembrol, isopimarinol, phytol, pimarinal, and sandaracopimarinol [55]. In contrast to branch bark, this source was dominated by oxygenated compounds, particularly diterpene alcohols, diols, acetates, aldehydes, and oxidized diterpenoids. Heartwood resin contained six compounds, namely 4-epiisocembrol, cembrene, epimanool, isocembrol, larixol, and larixyl acetate [40]. Finally, the neutral fraction of oleoresin contained only 13-epitorulosol [26]. Thus, branch bark and woody greens accounted for most of the documented diterpene diversity, but their profiles differed substantially in the degree of oxygenation.

Most constituents showed a restricted distribution among plant sources. Of the 40 unique compounds, 38 were reported from only one source, whereas only cembrene and isocembrol occurred in more than one source. Cembrene was shared between branch bark and heartwood resin [40,42], while isocembrol occurred in heartwood resin and woody greens [40,55]. No compound was reported from three or four sources. Moreover, the single oleoresin constituent, 13-epitorulosol, was not reported from the other materials [26]. The limited overlap distinguishes the hydrocarbon-rich branch bark profile from the oxygenated diterpene profiles of woody greens and heartwood resin. However, this pattern should be interpreted in the context of uneven analytical coverage, as each source was characterized in a separate study rather than through direct comparative profiling under standardized conditions.

Quantitative values were reported for 34 of the 42 records, although their availability differed markedly among sources. In branch bark essential oil, cembrene was the principal diterpene at 14.5%, followed by isopimaradiene at 2.31%, *cis*/*trans*-18-norabieta-3,8,11,13-tetraenes at 1.91%, *trans*-biformene at 1.61%, 18-norabieta-8,11,13-triene at 1.51%, and abietadiene at 1.31% [42]. The remaining branch bark diterpenes occurred at concentrations below 1%. Woody greens contained several quantitatively prominent oxygenated diterpenes, including 15-hydroxymethyldehydroabietate at 23.13%, dehydroabietinol at 10%, 13-epimanool at 8%, isopimarinol at 7.06%, phytol at 7%, and epitorulosol acetate at 5.45% of the essential oil [55]. Epitorulosol accounted for 43.4% of the polar alcohol fraction, but this value cannot be directly compared with total essential oil percentages because it was calculated using a different measurement basis [55].

In contrast, no quantitative concentrations were reported for the six heartwood resin compounds or for 13-epitorulosol in oleoresin [26,40]. Moreover, the apparent separation between hydrocarbon-rich branch bark and oxygenated woody greens may partly reflect differences in extraction, fractionation, and analytical targeting. The available evidence therefore demonstrates substantial structural diversity among *L. sibirica* diterpenes, but direct comparisons of tissue abundance remain constrained by source-specific studies and non-equivalent measurement bases.

#### 3.4.4. Resin Acids

Nine unique resin acids were reported from heartwood resin and needles, corresponding to 14 compound-source occurrences and 14 literature records from two publications (Table S3). Heartwood resin contained seven compounds: abietic, dehydroabietic, isopimaric, levopimaric, neoabietic, pimaric, and sandaracopimaric acids [40]. Needles also contained seven compounds, namely 15-hydroxyabietic, 15-hydroxydehydroabietic, abietic, dehydroabietic, isopimaric, levopimaric, and sandaracopimaric acids [46]. Thus, the two sources showed equal numerical diversity but differed in the occurrence of several source-specific constituents.

Five resin acids were shared between heartwood resin and needles. These included abietic, dehydroabietic, isopimaric, levopimaric, and sandaracopimaric acids [40,46]. In contrast, neoabietic and pimaric acids were reported only from heartwood resin [40], whereas 15-hydroxyabietic and 15-hydroxydehydroabietic acids were detected only in yellow needles [46]. Consequently, most of the resin acid profile was conserved across the two sources, while the hydroxylated derivatives were specifically associated with yellow needles in the available data. However, because each source was examined in a separate study, the absence of these compounds from other materials cannot be considered definitive evidence of tissue specificity.

The quantitative profile of heartwood resin was dominated by isopimaric acid, which accounted for 19.28% of total resinous compounds [40]. Abietic and sandaracopimaric acids were present at similar concentrations of 13.96% and 13.91%, respectively, followed by levopimaric acid at 10.86% and neoabietic acid at 10.18% [40]. Dehydroabietic acid represented 3.18% of total resinous compounds, while pimaric acid accounted for 1.26% of the resin hexane fraction [40]. Therefore, the heartwood resin profile was characterized by several abundant acids rather than by a single overwhelmingly dominant constituent.

Needles showed a different quantitative pattern. Dehydroabietic acid displayed the broadest and highest reported range, varying from 4.68% to 20.70%, whereas abietic acid ranged from 2.84% to 5.60% and isopimaric acid from 3.96% to 4.36% [46]. Sandaracopimaric and levopimaric acids occurred at lower levels of 0.57% to 0.74% and 0.24% to 0.35%, respectively [46]. In yellow needles, 15-hydroxyabietic and 15-hydroxydehydroabietic acids were reported at 3.34% and 3.66%, respectively [46]. The wide range observed for dehydroabietic acid indicates substantial variation within the needle samples, although the current data do not establish whether this variation reflects developmental condition, sample heterogeneity, or another source of variability.

All 14 records included quantitative values, making resin acids one of the most completely quantified phytochemical groups in the compiled dataset. Nevertheless, direct comparison between heartwood resin and needles remains limited because the values were calculated relative to different materials. Most heartwood values represent percentages of total resinous compounds, whereas the needle values describe the composition of needle samples; pimaric acid was additionally quantified relative to a resin hexane fraction [40,46]. Accordingly, the data support the presence of a shared resin acid core but do not provide a standardized basis for comparing absolute abundance between the two sources.

#### 3.4.5. Triterpenoids, Phytosterols, and Squalene

Fourteen unique triterpenoids and sterol-related compounds were reported from four plant sources, corresponding to 21 compound-source occurrences and 22 literature records from four publications (Table S3). Needles contained nine compounds: 24-methylenecycloartanol, 24-methylenelophenol, 24-methyllophenol, campesterol, citrastadienol, cycloartenol, cycloeucalenol, obtusifoliol, and β-sitosterol [47]. Cell cultures contained eight compounds: avenasterol, campesterol, cholesterol, cycloartenol, isofucosterol, squalene, stigmasterol, and β-sitosterol [52]. Woody greens contained 24-methylenecycloartanol, cycloartenol, and β-sitosterol [55], whereas branch bark contained only squalene [42]. Thus, needles and cell cultures accounted for most of the documented diversity, while woody greens and branch bark showed more restricted profiles.

Five compounds occurred in more than one source. Cycloartenol and β-sitosterol showed the broadest distributions, with both compounds reported from needles, cell cultures, and woody greens [47,52,55]. In addition, 24-methylenecycloartanol was shared between needles and woody greens [47,55], campesterol between needles and cell cultures [47,52], and squalene between branch bark and cell cultures [42,52]. The remaining nine compounds were reported from only one source. Specifically, 24-methylenelophenol, 24-methyllophenol, citrastadienol, cycloeucalenol, and obtusifoliol were restricted to needles [47], whereas avenasterol, cholesterol, isofucosterol, and stigmasterol were restricted to cell cultures [52]. This distribution indicates a shared core of cycloartenol-derived sterols accompanied by several source-associated intermediates and end products.

The needle extract contained comparatively high proportions of citrastadienol, β-sitosterol, cycloartenol, and 24-methylenecycloartanol at 7.69%, 7.18%, 6.82%, and 6.75%, respectively [47]. By contrast, 24-methyllophenol accounted for 1.84%, campesterol for 0.33%, 24-methylenelophenol for 0.10%, cycloeucalenol for 0.09%, and obtusifoliol for 0.01% of the total extract [47]. In woody greens, β-sitosterol represented 1.24% and cycloartenol 0.23% of the essential oil, while 24-methylenecycloartanol was detected only at trace level [55]. Squalene constituted 2.51% of branch bark essential oil [42]. Accordingly, the needle profile included several quantitatively prominent triterpenols and phytosterols, whereas the corresponding compounds occurred at lower reported levels in woody greens.

Cell cultures showed a different quantitative pattern. β-Sitosterol was the most abundant measured phytosterol, ranging from 410.0 to 1684.8 μg/g dry weight, followed by stigmasterol at 21.8 to 248.2 μg/g and campesterol at 102.4 to 199.2 μg/g [52]. Cholesterol was detected at substantially lower concentrations of 4.2 to 17.6 μg/g dry weight [52]. Isofucosterol accounted for 12.9% to 18.0% of total free sterols in embryogenic cell lines but was present at trace levels in one nonembryogenic line and at 2.6% in another [52]. Squalene varied from 2.3% to 34.2% of the total sterol ester fraction, whereas avenasterol and cycloartenol were identified without quantitative values [52]. These broad ranges demonstrate substantial heterogeneity among the analyzed cell lines, although the values cannot be converted directly into absolute differences without equivalent measurement units and reference fractions.

Quantitative information was available for 19 of the 22 records, but cross-source comparison remains constrained by incompatible measurement bases. Needle compounds were reported mainly as percentages of the total extract, cycloartenol was additionally expressed relative to unsaponifiable substances, woody green and branch bark compounds were measured in essential oils, and cell culture constituents were expressed as μg/g dry weight or as percentages of free sterols and sterol ester fractions [42,47,52,55]. Consequently, the reported values characterize composition within each analyzed fraction rather than absolute abundance across plant sources. The evidence nevertheless identifies needles and cell cultures as the most chemically diverse sources of triterpenoid and sterol-related constituents in the current literature.

### 3.5. Lipids and Related Compounds

#### 3.5.1. Fatty Acids

Twenty-one unique fatty acids were reported from needles, corresponding to 21 compound-source occurrences and 27 literature records from two publications (Table S4). The profile included 12 saturated fatty acids: lauric, myristic, palmitic, heptadecanoic, stearic, arachidic, behenic, tricosanoic, lignoceric, hexacosanoic, octacosanoic, and triacontanoic acids. In addition, 14-methylpalmitic acid represented the only branched saturated fatty acid, while (Z)-9-hexadecenoic acid was the only monounsaturated fatty acid. Seven polyunsaturated fatty acids were also identified: (Z)-7,10-hexadecadienoic, (Z)-7,10,13-hexadecatrienoic, (Z)-6,9,12-octadecatrienoic, linoleic, α-linolenic, (Z)-5,11,14,17-eicosatetraenoic, and (Z)-11,14,17-eicosatrienoic acids [46,48]. Thus, the needle fatty acid profile encompassed chain lengths from C12 to C30 and included saturated, monounsaturated, branched, and polyunsaturated constituents.

Quantitatively, α-linolenic acid was the predominant constituent, accounting for 22.46% to 40.68% of the total free and bound acids [46,48]. Palmitic acid was the principal saturated fatty acid at 15.06% to 17.71%, while linoleic acid ranged from 2.46% to 15.03% [46,48]. Several additional fatty acids showed intermediate maximum values, including myristic acid at up to 6.60%, (Z)-6,9,12-octadecatrienoic acid at up to 4.45%, triacontanoic acid at up to 3.59%, lignoceric acid at up to 2.96%, (Z)-7,10,13-hexadecatrienoic acid at up to 2.91%, and hexacosanoic acid at up to 2.60% [46,48]. By contrast, many long-chain saturated and unsaturated minor constituents occurred at concentrations below 2% or ranged from trace levels to low percentage values.

The predominance of α-linolenic, palmitic, and linoleic acids indicates that the documented profile was concentrated in a small number of major constituents, whereas the remaining fatty acids formed a chemically diverse minor fraction. However, the reported ranges combine free and bound acids and therefore do not distinguish between unesterified fatty acids and those released from complex lipids. Moreover, all 21 compounds were characterized exclusively in needles, leaving the fatty acid composition of bark, resin, wood, roots, reproductive tissues, and cell cultures unresolved. Although the database contains two publications [46,48], the identical compound lists and quantitative ranges suggest that these reports may represent closely related or corresponding versions of the same dataset rather than independent experimental confirmation. Consequently, the evidence provides detailed quantitative coverage of needle fatty acids but remains limited in tissue breadth and independent replication.

#### 3.5.2. Long-Chain Alcohols, Aldehydes, Ketones, and Wax Components

Four unique long-chain wax constituents were reported from needles and woody greens, corresponding to six compound-source occurrences and six literature records from two publications (Table S4). The needle wax fraction contained three secondary long-chain alcohols, namely nonacosan-10-ol, heptacosan-10-ol, and octacosan-10-ol, together with the long-chain ketone nonacosan-10-one [46]. Woody greens contained two of these compounds, nonacosan-10-ol and nonacosan-10-one [55]. Therefore, nonacosan-10-ol and nonacosan-10-one were shared between the two plant sources, whereas heptacosan-10-ol and octacosan-10-ol were reported only from needle wax.

The needle wax fraction was strongly dominated by nonacosan-10-ol, which accounted for 95.5% of the analyzed fraction [46]. Nonacosan-10-one represented 3.4%, while heptacosan-10-ol and octacosan-10-ol accounted for 0.8% and 0.3%, respectively [46]. Together, these four values account for the complete reported wax fraction, indicating a highly concentrated composition dominated by a single C29 secondary alcohol. In woody greens, nonacosan-10-ol represented 9.5% of the essential oil, whereas nonacosan-10-one accounted for only 0.34% [55]. Thus, nonacosan-10-ol was the predominant member of this compound group in both sources, although its reported proportion differed markedly.

All six records included quantitative values, but the results for needles and woody greens cannot be compared directly because they describe different chemical fractions. The needle values were calculated relative to an isolated wax fraction, whereas the woody green values were expressed relative to the essential oil [46,55]. Therefore, the lower percentages in woody greens do not necessarily indicate lower tissue concentrations. In addition, the absence of other long-chain alcohols and ketones from woody greens may reflect differences in extraction and analytical targeting. The current evidence nevertheless identifies nonacosan-10-ol as the dominant reported wax-related constituent of *L. sibirica*, while demonstrating that the broader diversity and tissue distribution of these compounds remain poorly characterized.

### 3.6. Prostaglandins

Six prostaglandins were identified in the cambial zone and buds of *L. sibirica*, corresponding to six unique compounds, six compound-source occurrences, and six literature records from a single publication (Table S1). The reported compounds comprised prostaglandins F_1_α and F_2_α, prostaglandins E_1_ and E_2_, prostaglandin A_2_, and prostaglandin B_2_ [35]. Thus, the profile included representatives of the F, E, A, and B prostaglandin series, all of which were detected in the same plant material. Unlike most other phytochemical groups examined in *L. sibirica*, these prostaglandins were reported exclusively from the cambial zone and buds and were not identified in bark, needles, wood, resin, flowers, or cell cultures [35]. However, this restricted distribution cannot be interpreted as evidence of tissue specificity because no comparable prostaglandin analyses were reported for other plant sources. Rather, it reflects the limited scope of the available evidence, which is based on a single investigation of one combined tissue source.

Furthermore, all six compounds were identified qualitatively, without reported concentrations or information on their relative abundance [35]. The absence of quantitative data prevents assessment of which prostaglandin series predominates in the cambial zone and buds. In addition, the lack of confirmation by independent studies limits evaluation of the reproducibility of these findings. Consequently, the available evidence establishes prostaglandins as a distinct but insufficiently characterized component of the reported *L. sibirica* phytochemical profile. Further targeted lipidomic analyses are required to confirm their occurrence, determine their concentrations, and establish their distribution across plant tissues.

### 3.7. Arabinogalactan

Arabinogalactan was the only individually characterized polysaccharide reported from *L. sibirica* in the compiled dataset. Two arabinogalactan fractions, designated arabinogalactan A and arabinogalactan B, were isolated from wood and represented 84.3% and 15.7% of the isolated polysaccharide fraction, respectively [33] (Table S1). Together, these fractions accounted for the complete reported polysaccharide fraction, with arabinogalactan A constituting the predominant component. Although the two fractions were entered separately in the database, they should not necessarily be interpreted as two structurally independent polysaccharides. Rather, the designations A and B refer to fractions obtained during the isolation procedure, and the available quantitative values describe their relative contributions to the recovered polysaccharide material [33]. Importantly, the values of 84.3% and 15.7% do not indicate the total arabinogalactan content of the original wood because they were calculated relative to the isolated fraction rather than to dry biomass.

The available evidence therefore confirms wood as a source of arabinogalactan and demonstrates a strongly uneven distribution between the two isolated fractions. However, this conclusion is based on a single publication and provides no comparative information for bark, needles, resin, roots, or reproductive tissues [33]. Furthermore, no individual low-molecular-weight carbohydrates or additional structurally defined polysaccharides were identified in the compiled dataset. Consequently, the carbohydrate composition of *L. sibirica* remains less comprehensively characterized than its phenolic and terpenoid profiles.

## 4. Extraction, Fractionation, and Isolation Strategies

The chemical diversity of *L. sibirica* requires extraction methods tailored to both the target compound class and the selected plant material. Polar polysaccharides, moderately polar phenolics, volatile terpenes, resin acids, and wax constituents differ substantially in solubility and thermal stability. Consequently, no single procedure can recover the complete phytochemical profile efficiently. Solvent composition, temperature, extraction time, particle size, tissue preparation, and subsequent fractionation must therefore be considered together to balance recovery, selectivity, compound stability, and process sustainability.

Aqueous ethanol provides a practical compromise for the simultaneous extraction of arabinogalactan and dihydroquercetin from wood. Levdansky et al. evaluated ethanol concentrations of 5% to 25%, extraction times of 0.5 to 4 h, and temperatures of 80 to 100 °C using comminuted wood with a particle size of 1 to 3 mm. The highest combined recovery was obtained with 15% aqueous ethanol at 90 to 100 °C for 2 h, yielding 18.0% arabinogalactan and 1.8% dihydroquercetin on a dry-weight basis. At this solvent composition, the high water content maintains polysaccharide solubility, while ethanol improves the recovery of the less polar flavonoid. Increasing the ethanol concentration above 20% reduced arabinogalactan recovery, whereas concentrations below 10% were less effective for dihydroquercetin extraction. Mechanical pretreatment by disk milling or extrusion further shortened extraction to approximately 30 min by increasing the accessible surface area and disrupting the wood matrix [51]. However, this gain in processing speed must be balanced against additional energy requirements and equipment costs.

Bark requires more selective fractionation because phenolic acids, flavonoids, catechins, tannins, suberin-derived compounds, and lignin occur within a chemically complex matrix. Chumbalov et al. used acetone extraction followed by bicarbonate treatment, ethyl acetate partitioning, and silica-gel chromatography to separate phenolic acids from flavonoids and catechins. This sequential procedure enabled the isolation of dihydroflavonols, flavonol glycosides, and flavan-3-ols, but its multiple solvent and purification steps reduced throughput and increased solvent consumption [25]. Barabash et al. instead combined aqueous ethanol extraction with alkaline treatment and acid hydrolysis to obtain broader compositional information on tannins, resins, phenolic acids, and polysaccharides [57]. Although suitable for analytical characterization, strong alkaline and acidic conditions can alter native structures and are therefore less appropriate when intact compounds are required for preparative or biological studies.

Soxhlet extraction followed by liquid–liquid partitioning offers broader recovery from mixed crown material. Tranchuk et al. extracted needles, twigs, and small branches with isopropanol for 8 h and subsequently partitioned the extract using petroleum ether, diethyl ether, and ethyl acetate. The resulting fractions separated predominantly lipophilic constituents from less polar and more polar phenolics, thereby generating chemically differentiated extracts for subsequent characterization [44]. Nevertheless, prolonged heating and large solvent volumes limit the environmental and practical advantages of this method. Moreover, solvent fractions should not be interpreted as chemically exclusive because compounds with intermediate polarity may distribute across more than one phase.

Oleoresin and essential oils require different strategies because their principal constituents are volatile or strongly lipophilic. Khan et al. fractionated 3 kg of oleoresin by vacuum distillation and silica-gel chromatography, recovering 480 g of monoterpene hydrocarbons, 13.2 g of sesquiterpene hydrocarbons, and 12 g of monoterpene alcohols. Reduced-pressure distillation limited exposure to high temperatures, while chromatographic gradients separated hydrocarbon and oxygenated fractions [31]. Despite providing chemically defined fractions, the procedure required a large quantity of starting material and several laborious purification steps. Preparative gas chromatography and high-performance liquid chromatography may improve resolution for selected constituents, but their suitability for large-scale processing depends on compound volatility, sample complexity, and production cost.

Steam distillation was the principal preparative method used to isolate essential oils from *L. sibirica* woody verdure and twigs (Table 1). In the earlier study, woody verdure was subjected to steam distillation, after which the recovered essential oil was fractionated and characterized by gas–liquid chromatography, infrared spectroscopy, and thin-layer chromatography [32]. A later investigation applied steam distillation under atmospheric pressure to freshly collected twigs from natural populations and analyzed 50 resulting essential oil samples using GC-MS, GC-FID, chiral comprehensive two-dimensional gas chromatography, and NMR spectroscopy [49]. This combination of preparative isolation and complementary analytical methods enabled detailed compositional characterization and assessment of geographical variation [49]. By contrast, volatile compounds from female flowers were collected by dynamic headspace sampling on TENAX GC, eluted with *n*-pentane, and analyzed by GC-MS [34]. This method characterizes emitted volatiles without producing a preparative essential oil and should therefore be distinguished from steam distillation. Although steam distillation provides sufficient material for fractionation and structural analysis, operation at atmospheric pressure exposes volatile constituents to heat and may not fully preserve thermolabile compounds. However, the available studies did not directly compare steam distillation with lower-temperature methods or report compound-specific recovery. Consequently, steam distillation remains the only clearly documented preparative method for *L. sibirica* essential oils in the reviewed literature, while comparative evidence on extraction yield, thermal stability, and method-dependent composition remains limited.

The effectiveness of extraction is influenced by both plant source and harvest condition. Seasonal investigations of *L. sibirica* included comparative analyses of oleoresin monoterpenes [27], prostaglandins from the cambial zone and buds [35], glycolipids from woody verdure [36], and extractives from crown tissues and needles [44,46,48]. Crown material comprising needles, twig bark, woody portions of twigs, and fallen needles was collected in summer and autumn and subjected to sequential isopropanol extraction followed by petroleum ether, diethyl ether, and ethyl acetate fractionation [44]. Similarly, green and yellow needles were analyzed by sequential solvent extraction and separation into neutral, acidic, resinous, and wax fractions [46,48]. These studies demonstrate that harvest condition was treated as an important determinant of extract composition. However, differences in sample preparation and fraction definition prevent a unified quantitative comparison of seasonal extraction efficiency. Accordingly, harvest timing should be optimized for a specified compound class and extraction procedure rather than on the basis of total extract yield alone.

Plant source selection is equally important because the reported phytochemical groups were distributed unevenly among *L. sibirica* tissues. Bark yielded flavonols, dihydroflavonols, flavonol glycosides, catechins, procyanidins, phenolic acids, and condensed tannins following acetone, methanol, ether, or sequential solvent extraction [25,28,29,37]. By contrast, knotwood provided taxifolin, lignans, and oligolignans after aqueous isopropanol or acetone extraction and chromatographic purification [41,53]. Wood was used for the isolation of arabinogalactan and dihydroquercetin through cold-water extraction or aqueous ethanol extraction [33,51]. Needles contained a broader mixture of flavonoid glycosides, fatty acids, resin acids, triterpenoids, phytosterols, and wax constituents [30,46,47,48], whereas oleoresin and heartwood resin were particularly rich in mono-, sesqui-, and diterpenes as well as resin acids [26,27,31,40]. Thus, tissue selection should be based on the target chemical class. Nevertheless, these patterns reflect the available extraction studies and should not be interpreted as standardized comparisons of absolute phytochemical abundance among tissues.

The reviewed literature also reveals a strong dependence on conventional extraction and fractionation procedures. These included solvent maceration, sequential liquid–liquid extraction, Soxhlet extraction, steam distillation, acid-base fractionation, precipitation, and column chromatography (Table 1). More advanced techniques were used mainly for downstream analysis and purification rather than for primary extraction. For example, supercritical fluid chromatography was applied to the analytical and semi-preparative separation of knotwood lignans after conventional hexane de-resination and acetone extraction [53]. It should not be confused with supercritical carbon dioxide extraction, which was not reported as a primary extraction method in the included studies. Therefore, the available evidence does not support direct conclusions regarding the efficiency of supercritical fluid extraction for *L. sibirica*.

## 5. Biological Activities and Preliminary Medical Applications

### 5.1. Antioxidant Activity

Antioxidant activity is among the most extensively investigated biological properties of *L. sibirica* phytochemicals. The evidence is concentrated primarily on taxifolin, phenolic acids, and lignans and is derived mainly from chemical oxidation and radical-scavenging assays. These studies support direct radical interception as the best-established mechanism, whereas metal chelation, modulation of endogenous antioxidant enzymes, and activation of cellular signaling pathways remain less directly demonstrated for compounds isolated specifically from *L. sibirica*. Accordingly, chemical antioxidant capacity should be distinguished from antioxidant efficacy in biological systems. Taxifolin has received particular attention because it is a prominent flavonoid in *L. sibirica* bark and woody tissues. In a model of radical-chain oxidation of propan-2-ol, taxifolin showed the highest antioxidant activity among the natural compounds evaluated [58]. Its radical-scavenging capacity is associated principally with the 3′,4′-dihydroxy substitution pattern in the B ring, which enables hydrogen donation to peroxyl radicals and stabilizes the resulting phenoxyl radical through electron delocalization [58,59]. Consistent with this interpretation, dihydrokaempferol, which lacks the 3′-hydroxyl group, showed lower activity than taxifolin [58]. The 3-hydroxyl and 4-carbonyl groups in the C ring may further contribute to radical stabilization and provide potential coordination sites for transition metals. However, the relative contribution of each structural feature depends on the assay system, and the ranking observed in propan-2-ol oxidation should not be generalized to all biological or chemical environments. In addition to radical scavenging, taxifolin has been proposed to reduce oxidative reactions through metal chelation. The 3-hydroxyl and 4-carbonyl groups, together with the 5-hydroxyl and 4-carbonyl arrangement, can potentially coordinate iron and copper ions [59]. Such interactions could limit metal-catalysed formation of hydroxyl radicals and thereby reduce lipid, protein, or nucleic acid oxidation. Nevertheless, the available *L. sibirica* studies primarily demonstrate antioxidant activity in oxidation models rather than direct measurements of metal-binding affinity or inhibition of Fenton chemistry. Metal chelation should therefore be considered a structurally plausible mechanism rather than a fully established mechanism in this species.

Phenolic acids from *L. sibirica* bark also showed structure-dependent antioxidant activity. Among caffeic, ferulic, vanillic, and 4-hydroxybenzoic acids, caffeic acid exhibited the strongest activity [58]. This result is consistent with its adjacent hydroxyl groups, which facilitate hydrogen donation and stabilization of the resulting radical. Ferulic acid showed intermediate activity, reflecting the combined effects of its hydroxyl group, methoxy substituent, and conjugated side chain. By contrast, vanillic and 4-hydroxybenzoic acids showed lower activity, consistent with their less extensive hydroxylation and conjugation [58,60]. Thus, the reported activity generally increased with the capacity of the aromatic structure to stabilize radical intermediates. However, these relationships were established in chemical assays and do not account for differences in solubility, metabolism, or cellular uptake.

Lignans provide a second major class of antioxidant constituents. Secoisolariciresinol, isolariciresinol, and lariciresinol showed substantial activity in single-electron-transfer and superoxide-scavenging assays [20,23]. Their activity has been attributed to hydrogen donation from phenolic hydroxyl groups and stabilization of the resulting phenoxyl radicals. In some assay systems, lignans showed activity comparable to or greater than that of taxifolin [23]. However, this apparent superiority was assay-dependent and should not be interpreted as evidence that lignans are universally more effective antioxidants. Differences in reaction mechanism, radical species, solvent, concentration, and endpoint can alter the relative ranking of phenolic compounds. Direct comparison therefore requires evaluation under identical experimental conditions and across complementary antioxidant assays.

### 5.2. Antiviral Activity

Antiviral evidence for *L. sibirica* is centred almost entirely on dihydroquercetin (taxifolin) and Coxsackievirus B4 (CVB4), rather than on chemically complex extracts or a broad range of viral pathogens. In Vero cells, dihydroquercetin at 100 μg/mL reduced the CVB4 titre and virus-induced cytopathic effects relative to the infected control [61]. Time-of-addition experiments showed the greatest activity when treatment was applied during the first 1–3 h after infection, indicating interference with an early stage of viral reproduction [62]. However, the specific molecular target was not identified, and the available results do not distinguish between direct inhibition of a viral component and indirect protection of host cells.

The activity observed in cell culture was subsequently examined in mice with CVB4-induced pancreatitis using dihydroquercetin isolated from *L. sibirica* heartwood. Intraperitoneal administration at 75 or 150 mg/kg/day for five days produced a dose-dependent reduction in pancreatic viral titres, with the higher dose decreasing the titre by approximately 2.4 log units on day 5 after infection. Dihydroquercetin also reduced inflammatory infiltration, the number of pancreatic inflammatory foci and tissue destruction, while restoring pancreatic antioxidant activity to a level close to that of uninfected animals [63]. At 150 mg/kg, its antiviral and histological effects were comparable to those of ribavirin, although the differences between the two treatments were not statistically significant for the morphometric outcomes [62]. These findings extend the antiviral evidence from an isolated cell system to an animal model and suggest that viral suppression may occur together with attenuation of oxidative and inflammatory tissue injury.

Nevertheless, the evidence remains preliminary. Both primary studies investigated the same compound against the same viral strain, and the animal experiment included only five mice per treatment group, used intraperitoneal administration and assessed outcomes at a single principal time point [61,62]. Moreover, restoration of antioxidant activity and reduced pancreatic inflammation cannot establish whether dihydroquercetin acts directly on CVB4 replication or indirectly through host-protective effects. Quercetin and its derivatives have demonstrated activity against multiple viral families through effects on viral entry, replication-associated proteases and infection-induced inflammation, but these broader mechanisms are derived from studies of flavonoids in general and cannot be assigned specifically to *L. sibirica* dihydroquercetin without direct experimental confirmation [63]. Accordingly, further studies should identify the molecular target, determine selectivity and cytotoxicity, evaluate pharmacokinetics and oral bioavailability, and test additional enteroviruses and clinically relevant infection models before therapeutic conclusions are drawn.

### 5.3. Antibacterial Activity

Polyphenolic compounds can affect microbial physiology through interactions with cellular membranes, proteins and nucleic acids, extending their biological activity beyond conventional antioxidant effects [64]. However, direct antibacterial evidence for *L. sibirica* remains limited. The most detailed mechanistic study evaluated a procyanidin-rich preparation obtained from the bark of *Larix gmelinii*, rather than *L. sibirica* [65]. The findings therefore provide comparative evidence for the antibacterial potential of larch bark procyanidins, but they should not be interpreted as direct confirmation of equivalent activity in *L. sibirica*.

The larch bark procyanidin preparation inhibited the growth of *Staphylococcus aureus* CGMCC 1.8721 in vitro, with a minimum inhibitory concentration of 1.75 mg/mL [65]. Growth-curve analysis at one-half, three-quarters and the full minimum inhibitory concentration demonstrated concentration-dependent growth suppression. However, the study did not report a minimum bactericidal concentration or a standardized time-kill analysis. Consequently, the results establish growth inhibition but do not conclusively demonstrate that the preparation was bactericidal. Moreover, the relatively high minimum inhibitory concentration and the absence of comparisons with conventional antibiotics limit assessment of its antibacterial potency.

Membrane and cell-wall disruption appeared to represent the principal antibacterial effect. Scanning and transmission electron microscopy showed surface deformation, irregular protrusions, extracellular material and progressive loss of envelope integrity after treatment with 1.75 mg/mL of the procyanidin preparation. These morphological changes were supported by increased extracellular alkaline phosphatase and β-galactosidase activities, together with increased conductivity of the bacterial suspension, indicating leakage of enzymes and ions through a compromised cellular envelope [65]. Similar combinations of microscopy, conductivity and intracellular-component leakage have been used to evaluate membrane damage caused by other plant-derived antibacterial compounds [66]. Nevertheless, these measurements are indirect indicators of permeability and do not identify a specific membrane or cell-wall target.

Disruption of the bacterial envelope was accompanied by changes in ion regulation and energy metabolism. Treatment increased Na^+^/K^+^-ATPase and Ca^2+^-ATPase activities, which may reflect a compensatory response to altered ionic homeostasis, whereas succinate dehydrogenase, malate dehydrogenase and total ATPase activities decreased after prolonged exposure [65]. The largest reported reduction was observed for succinate dehydrogenase, which declined by 13.8% after 8 h, while reductions in malate dehydrogenase and total ATPase were comparatively modest. SDS-PAGE also revealed changes in the intensity and number of bacterial protein bands after 6 and 24 h [65]. However, these observations demonstrate alterations in the total protein profile rather than direct inhibition of protein synthesis [66]. Because membrane damage can produce widespread secondary metabolic changes, the enzymatic and protein alterations cannot be regarded as independent molecular targets without targeted proteomic or biochemical validation.

A possible interaction with bacterial DNA was inferred from fluorescence quenching, ultraviolet absorption, ionic-strength experiments and competition with the minor-groove probe DAPI [6]. The spectroscopic patterns were interpreted as static complex formation and possible minor-groove binding, following analytical approaches previously applied to interactions between small molecules and isolated DNA [67,68]. However, these experiments used purified *S. aureus* genomic DNA outside intact bacterial cells and therefore do not demonstrate that procyanidins reached or bound bacterial DNA under physiological treatment conditions. Overall, the study supports a multicomponent response dominated by cell-envelope damage, followed by impaired ionic balance and metabolic function, whereas effects on protein expression and DNA remain mechanistic hypotheses [65]. Further studies should chemically standardize the procyanidin preparation, distinguish bacteriostatic from bactericidal activity, evaluate resistant clinical isolates and biofilms, and directly compare procyanidins from authenticated *L. sibirica* and *L. gmelinii* bark.

### 5.4. Neuroprotective and Neuritogenic Activity

The neuroactive effects of *L. sibirica* phenolics have been investigated using three compounds isolated from knotwood: (+)-dihydroquercetin (taxifolin), (−)-secoisolariciresinol and (+)-isolariciresinol. The compounds were purified to greater than 99% chemical and optical purity and evaluated in primary neuronal and glial cultures obtained from embryonic or early postnatal mice [41]. Rather than measuring protection against a defined neurotoxic insult, the study examined neurite extension, neuronal migration, Schwann cell process formation and astrocyte migration. Thus, the evidence primarily demonstrates neuritogenic and cell-modulatory activity, whereas direct neuroprotection against neuronal injury or degeneration was not established.

All three compounds promoted neurite outgrowth at concentrations ranging from 100 pM to 1 μM, although their effective concentration ranges differed among neuronal populations. In cerebellar granule neurons, maximal stimulation reached approximately 1.6-fold relative to the vehicle control at 100 nM. Dorsal root ganglion neurons treated with 100 nM of each compound showed an approximately 1.7-fold increase in neurite length. The strongest response was observed in embryonic motoneurons, in which maximal neurite outgrowth increased by 1.9-fold with (+)-isolariciresinol, 2.7-fold with (−)-secoisolariciresinol and 3.1-fold with (+)-dihydroquercetin [41]. However, the motoneuron response was not maintained at 500 nM, at which neurite growth returned to the control level, indicating a non-linear concentration-response relationship. By comparison, cardanol increased the growth of immortalized motoneuron-like NSC-34 cells by approximately 1.4-fold, whereas selected curcuminoids increased neurite length in PC12 cells by approximately 1.4- to 2.2-fold at micromolar concentrations [69,70]. Although the *L. sibirica* compounds were active at lower concentrations, differences in cell type, culture conditions and outcome measurement preclude a direct potency ranking across these studies.

The phenolic compounds also affected neuronal migration and peripheral glial morphology. At 100 nM, (−)-secoisolariciresinol and (+)-isolariciresinol increased the total number of cells migrating from cerebellar explants by approximately 1.2-fold, whereas (+)-dihydroquercetin produced a 1.4-fold increase. In vehicle-treated explants, only 3% of migrating cells travelled more than 301 μm from the explant margin. This proportion increased to 8% following treatment with either lignan and to 12% following treatment with (+)-dihydroquercetin, indicating that taxifolin exerted the strongest effect on migration distance [41]. In addition, all three compounds promoted Schwann cell process formation between 100 pM and 100 nM. Maximum process length increased by 1.8-fold with the two lignans and by 1.9-fold with (+)-dihydroquercetin [41]. These responses are consistent with previous evidence that quercetin at 0.3–33 μM can support Schwann cell survival and outgrowth and promote peripheral nerve regeneration in a rat nerve-gap model [71]. Nevertheless, increased process length in isolated Schwann cells does not by itself demonstrate enhanced myelination, axonal guidance or functional nerve repair.

In contrast to their stimulatory effects on neurons and Schwann cells, the three compounds inhibited astrocyte migration after scratch injury. After 48 h, control astrocytes had migrated approximately 700 μm and closed 85% of the original gap, whereas treated astrocytes migrated only 500–550 μm, leaving 35–40% of the gap unclosed [41]. This cell-type-dependent response suggests that the compounds do not simply produce a general increase in cell viability or motility. Other polyphenols can also modulate glial behaviour, although their effects are not uniform. For example, resveratrol increased astroglial secretion of brain-derived neurotrophic factor by approximately five-fold and glial cell line-derived neurotrophic factor by up to six-fold through ERK1/2- and CREB-associated signalling [72], whereas epigallocatechin gallate inhibited the adhesion and migration of neural progenitor cells [73]. Accordingly, phenolic structure, concentration and cellular context appear to determine whether migration is enhanced or suppressed.

The inhibition of astrocyte migration was interpreted as potentially beneficial because excessive astrocyte recruitment can contribute to glial-barrier formation after central nervous system injury. However, a scratch-closure assay does not reproduce the cellular complexity of astrogliosis or establish that reduced migration would improve axonal regeneration in vivo. Moreover, Loers et al. did not identify the molecular receptors or signalling pathways responsible for neurite extension and migration, nor did they determine whether these responses resulted from antioxidant activity, altered cytoskeletal dynamics or neurotrophic signalling [41]. The study was further limited to short-term murine cell cultures, with outcomes assessed after 24–48 h and without pharmacokinetic, blood–brain barrier or functional behavioural data. Therefore, the findings identify (+)-dihydroquercetin, (−)-secoisolariciresinol and (+)-isolariciresinol as promising neuritogenic compounds, but their therapeutic relevance requires confirmation in models of peripheral nerve injury, spinal cord injury and neurodegenerative disease.

### 5.5. Anti-Inflammatory and Anti-Allergic Activities

Mast cells are central effector cells in immediate IgE-mediated allergic reactions [74]. Antigen-induced cross-linking of IgE-bound FcεRI initiates a tyrosine kinase signalling cascade that promotes degranulation and the release of histamine, lipid mediators and inflammatory cytokines [75,76]. Kim et al. evaluated whether a bark extract of *L. sibirica* and nine phenolic constituents could interfere with this response [74]. The experiments used IgE-sensitized rat basophilic leukaemia RBL-2H3 cells, a widely employed model of mast cell activation, although their basophilic origin and phenotypic differences from primary human mast cells must be considered when interpreting the results [77].

The *L. sibirica* bark material was extracted with 95% ethanol for three days, producing 123 g of dried extract from 1 kg of plant material, equivalent to an extraction yield of 12.3% [74]. The extract was tested at 1, 5 and 10 µg/mL, whereas dihydroquercetin, dihydrokaempferol, quercetin, kaempferol, eriodictyol, (+)-catechin, naringenin, resveratrol and secoisolariciresinol were evaluated predominantly at 20 µM. The authors reported no reduction in cell viability after 8 h of exposure to 5–40 μg/mL of the extract or 5–40 μM of the individual compounds [74]. Importantly, these compounds were purchased as analytical standards rather than isolated from the tested extract, and their concentrations in the extract were not determined. Consequently, their individual effects demonstrate the potential activity of compounds associated with *L. sibirica*, but they do not establish which constituents accounted for the activity of the complete extract.

The extract inhibited mast cell degranulation in a concentration-dependent manner. At 10 µg/mL, β-hexosaminidase release was reduced to approximately one-half of the antigen-stimulated control, while histamine release decreased from approximately 160 to 115 ng/mL [74]. Among the individual compounds, quercetin and kaempferol produced the strongest inhibition of both β-hexosaminidase and histamine release, whereas their saturated analogues, dihydroquercetin and dihydrokaempferol, were markedly less active [74]. Eriodictyol, (+)-catechin, naringenin, resveratrol and secoisolariciresinol displayed intermediate or outcome-dependent activity. These observations are consistent with previous studies showing that quercetin and kaempferol inhibit IgE-mediated mast cell activation and passive cutaneous anaphylaxis [78], while resveratrol and eriodictyol suppress mast cell degranulation in related experimental systems [79,80].

The differences among structurally related flavonoids suggest that the unsaturated C2-C3 bond of flavonols may contribute to anti-allergic activity. Quercetin was more active than dihydroquercetin, and kaempferol was more active than dihydrokaempferol [74]. Furthermore, quercetin and eriodictyol, which contain an additional hydroxyl group on the B ring, generally showed stronger activity than kaempferol and naringenin, respectively. Previous structure–activity studies similarly identified the C2-C3 double bond and the number and position of hydroxyl substituents as determinants of flavonoid-mediated inhibition of mast cell degranulation [81]. Nevertheless, Kim et al. compared the individual compounds primarily at a single concentration of 20 µM [74]. Therefore, the proposed structure–activity relationship remains preliminary because compound-specific concentration–response curves and IC_50_ values were not determined.

In addition to inhibiting the release of preformed granule contents, the extract suppressed newly synthesized inflammatory mediators. Antigen stimulation increased prostaglandin D2 release to approximately 1600 pg/mL, whereas treatment with 10 µg/mL of the extract reduced it to approximately 600 pg/mL. This response was accompanied by lower cyclooxygenase-2 expression. The extract also reduced the release and mRNA expression of interleukin-4 and tumour necrosis factor-α, and several individual phenolics produced similar effects at 20 µM [74]. Because interleukin-4 promotes IgE-associated type 2 immune responses and tumour necrosis factor-α contributes to inflammatory cell recruitment, simultaneous inhibition of these mediators suggests an effect extending beyond acute degranulation. However, mRNA levels were evaluated by semiquantitative RT-PCR rather than quantitative PCR, which limits the precision of the reported transcriptional changes.

Mechanistically, the extract and several phenolic standards reduced phosphorylation of Syk, PLCγ and PKCµ, followed by decreased activation of ERK, JNK and p38 MAPKs [74]. They also reduced IKKα/β phosphorylation and prevented IκB degradation, indicating attenuation of NF-κB signalling. This pattern is consistent with interference in the FcεRI-associated signalling network, in which Syk activation connects receptor cross-linking to calcium mobilization, degranulation and cytokine production [76]. Quercetin has independently been shown to inhibit NF-κB recruitment to pro-inflammatory gene promoters, whereas dihydroquercetin was less effective in the same experimental system [82].

Nevertheless, the immunoblotting results reported by Kim et al. showed changes in the activation of several signalling pathways but did not identify a direct molecular target [74]. The reduced phosphorylation of multiple downstream proteins may reflect inhibition of an upstream kinase, altered membrane-associated signalling or broader suppression of mast cell activation.

The cellular findings were supported by an IgE-mediated passive cutaneous anaphylaxis model in mice. Oral administration of the extract at 50 or 200 mg/kg one hour before antigen challenge reduced Evans blue extravasation, with the 200 mg/kg dose producing an approximately 43% reduction relative to the sensitized control [74]. Cetirizine at 20 mg/kg served as the reference treatment. Thus, orally administered extract exerted measurable activity in a local vascular model of immediate hypersensitivity. However, the experiment evaluated prophylactic administration in passive cutaneous anaphylaxis, rather than systemic anaphylaxis or established allergic disease. Furthermore, individual phenolics were not evaluated in vivo, the number of animals was not clearly reported, and pharmacokinetic exposure was not measured. The available evidence therefore supports mast-cell-stabilizing and anti-inflammatory activity of the *L. sibirica* bark extract, but confirmation in primary human mast cells, chronic models of allergic inflammation and chemically standardized extracts is required before therapeutic relevance can be established.

### 5.6. Immunomodulatory and Gut-Related Effects

**Figure 4:**
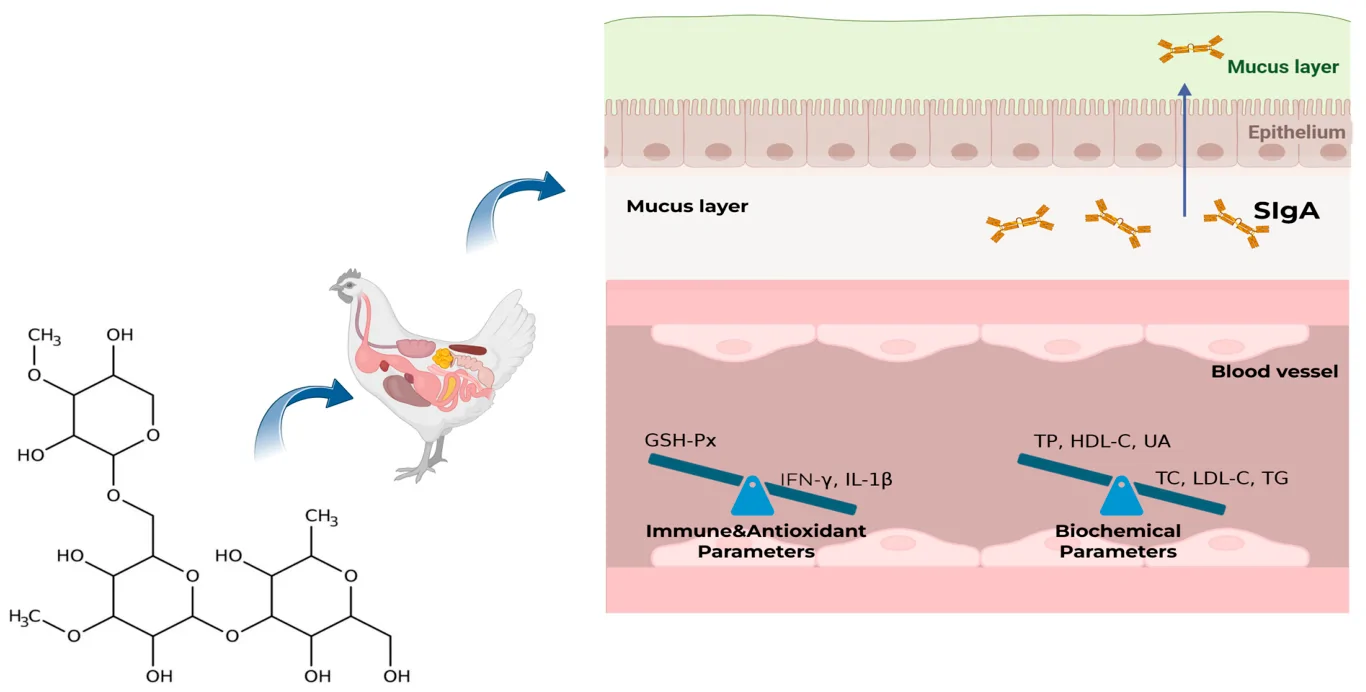
Schematic summary of the gut-related and systemic responses to dietary *L. sibirica* arabinogalactan in broilers. Blue curved arrows indicate dietary administration and the transition from the whole animal to the enlarged intestinal view; they do not represent experimentally demonstrated transport or causal pathways. In the lower panel, the balances summarize changes in measured markers: GSH-Px, total protein (TP), HDL-C and uric acid (UA) increased, whereas IFN-γ, IL-1β, total cholesterol (TC), LDL-C and triglycerides (TG) decreased. IL-10 and secretory IgA (sIgA) did not change significantly. The illustration is a schematic interpretation of the reported results, not an original experimental image [83].

Liu et al. examined the effects of dietary *L. sibirica* arabinogalactan in 160 male broilers assigned to a basal diet or the same diet supplemented with 25 mg/kg arabinogalactan for 42 days. The tested polysaccharide had an average molecular weight of 19.8 kDa and consisted predominantly of galactose, approximately 86.5–87.5%, and arabinose, approximately 9.6–10.6%, with arabinose-to-galactose ratios of 1:8.98 and 1:8.24 in two compositional analyses. Methylation analysis indicated a highly branched structure containing terminal Galp, 1,6-linked Galp and 1,3,6-linked Galp residues at 32.85%, 23.71% and 30.39%, respectively (Figure 4) [83]. These structural features are relevant because the molecular weight, branching pattern and side-chain architecture of polysaccharides may influence their fermentability and interactions with immune cells [84]. However, the occurrence of minor differences between the two monosaccharide analyses, together with the detection of trace constituents, indicates that the preparation was not chemically homogeneous. Consequently, the biological effects cannot be attributed to a single precisely defined arabinogalactan structure without further fractionation and activity-guided testing.

Dietary arabinogalactan increased final body weight, average daily gain, breast-muscle yield and leg-muscle yield relative to the control treatment (all *p* < 0.05), whereas feed intake was unaffected and the reduction in feed conversion ratio did not reach statistical significance (*p* = 0.115). Liu et al. also observed higher serum HDL cholesterol and total protein, together with lower triglyceride, total cholesterol and LDL cholesterol concentrations [83]. Although these changes suggest altered nutrient and lipid metabolism, the simultaneous increases in serum urea and uric acid should not automatically be interpreted as beneficial because they may reflect changes in protein turnover, nitrogen utilization or purine metabolism. Moreover, only one supplementation dose was tested. Thus, the experiment establishes an association between 25 mg/kg arabinogalactan and selected productive and metabolic outcomes, but it does not define a minimum effective dose, an optimal dose or the possibility of nonlinear responses.

The immunological results indicate selective modulation rather than generalized immune stimulation [85,86,87,88,89,90]. Arabinogalactan supplementation reduced serum IL-1β and IFN-γ concentrations (*p* < 0.001) and increased glutathione peroxidase activity (*p* < 0.001), whereas IL-10, globulin and total antioxidant capacity remained unchanged. Furthermore, higher duodenal villus height and a higher jejunal villus-height-to-crypt-depth ratio were accompanied by increased expression of occludin and the intestinal stem-cell marker *Lgr5* in the jejunum and cecum (*p* < 0.05). These findings are consistent with the preservation of epithelial integrity, particularly because occludin contributes to tight-junction organization. Previous experiments provide complementary evidence: Daguet et al. found that arabinogalactan-containing preparations improved barrier-associated responses in an in vitro human colonic ecosystem [85], whereas Zheng et al. reported protection against lipopolysaccharide-induced barrier injury in Caco-2 cells through signalling associated with AMPK, SIRT1 and NF-κB [89]. Nevertheless, Liu et al. measured gene expression rather than epithelial permeability directly, and the absence of changes in cecal secretory IgA, trypsin and chymotrypsin limits claims of broad enhancement of mucosal immunity or digestive function [83].

The intestinal effects were accompanied by changes in the cecal microbial community. Liu et al. reported higher Sobs, Ace and Chao richness indices (*p* < 0.001), but neither Shannon nor Simpson diversity differed between groups [83]. Accordingly, arabinogalactan increased the number of detected taxa without demonstrating a corresponding change in community evenness. At the genus level, supplementation increased the relative abundance of *Barnesiella*, the *Christensenellaceae R-7 group* and *Fournierella*, while the control microbiota showed greater representation of *Bacteroides*, *Phascolarctobacterium*, *Desulfovibrio* and *Enterorhabdus*. Cecal acetate and butyrate concentrations also increased (*p* < 0.05), whereas propionate and valerate were unaffected [83]. These observations agree with earlier evidence that arabinogalactan can be fermented by intestinal microorganisms and alter microbial or fermentation profiles in humans, dogs and in vitro systems [86,87,88]. For example, Robinson et al. found that 15 and 30 g/day of larch arabinogalactan increased total fecal anaerobes and reduced fecal ammonia in healthy adults, although short-chain fatty acids, transit time and blood lipids did not change significantly [86]. Such differences indicate that arabinogalactan responses depend on the host, dose, preparation and baseline microbiota.

Untargeted metabolomics detected 500 metabolites in positive-ion mode and 195 in negative-ion mode, of which eight increased and 26 decreased following supplementation [83]. The associated pathways included pantothenate and CoA biosynthesis, β-alanine metabolism, pyrimidine metabolism and ABC transporters [83]. In parallel, increased cecal expression of *FOXP3* and *TGF-β* suggests a local regulatory immune response that may be related to microbial fermentation products. However, pathway enrichment identifies statistical associations rather than biochemical flux, and the 16S rRNA analysis cannot establish whether the altered taxa produced the additional acetate and butyrate or caused the barrier and immune responses. Furthermore, changes in relative abundance do not necessarily represent changes in absolute bacterial numbers. Mechanistic confirmation would require targeted metabolite quantification, microbial absolute-abundance measurements, antibiotic-depletion or microbiota-transfer experiments and direct examination of receptor-mediated signalling.

Overall, Liu et al. provide integrated evidence linking *L. sibirica* arabinogalactan supplementation with growth, intestinal morphology, barrier-associated gene expression, microbial composition and selected immune markers in broilers [83]. Nevertheless, the conclusions are restricted by the single dose, 42-day duration, use of only male broilers and absence of a pathogen-challenge model. Evidence from dogs, human fermentation systems and clinical studies indicates that larch arabinogalactan can influence microbial or immune outcomes [85,86,87,88,91], but these findings are not directly interchangeable because the arabinogalactan preparations, doses and biological models differ. Notably, Riede et al. reported a reduction in the number of participants experiencing common-cold episodes after 4.5 g/day of a proprietary larch arabinogalactan preparation for 12 weeks, although the difference in cold incidence in the full analysis population was only borderline significant (*p* = 0.055) [91]. Therefore, the broiler study supports the biological activity of dietary *L. sibirica* arabinogalactan but does not by itself establish clinical efficacy or a defined mechanism in humans.

### 5.7. Anticancer Activity and Drug-Delivery Applications

**Figure 5:**
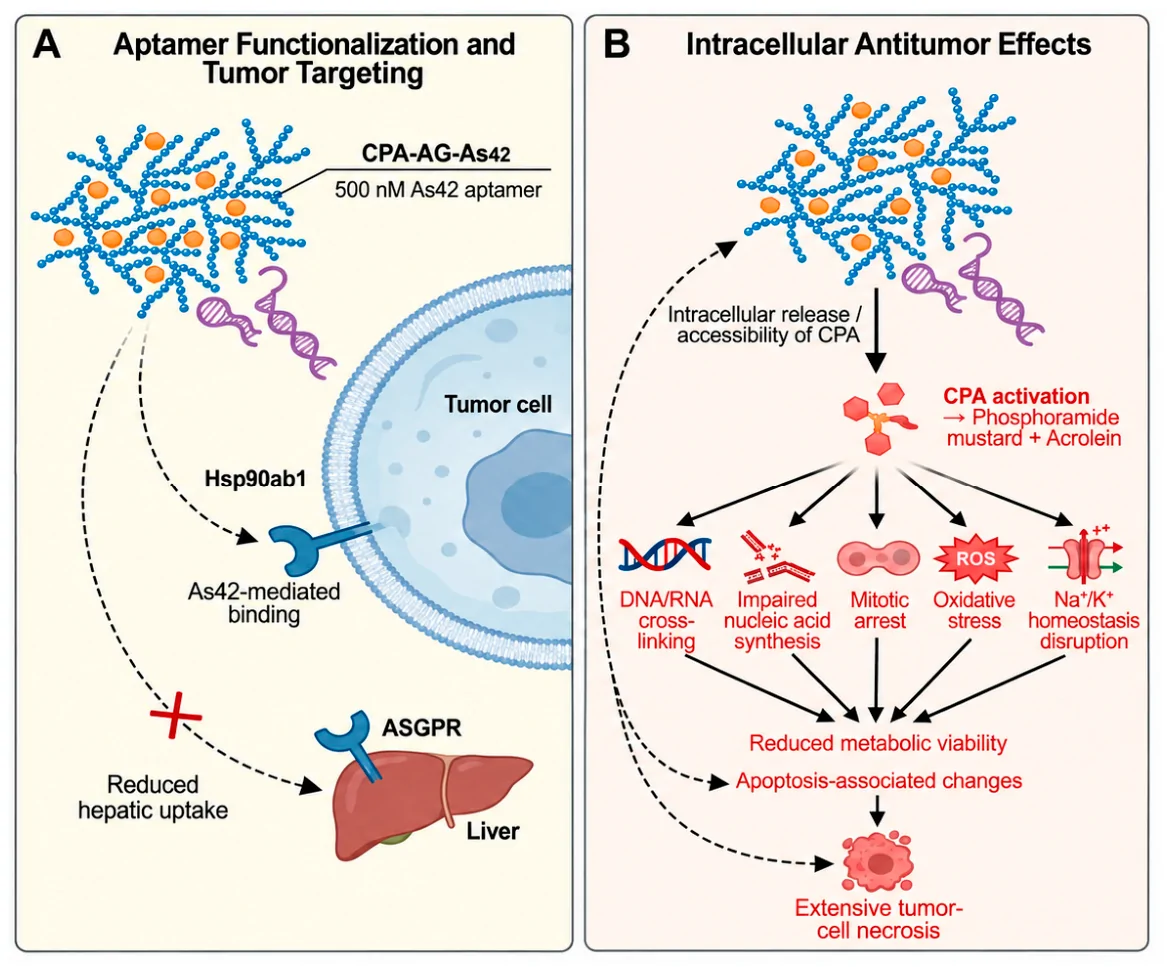
Proposed tumor targeting and antitumor effects of the As42-functionalized cyclophosphamide–*Larix sibirica* arabinogalactan formulation. (**A**) As42 functionalization and proposed binding to tumor-associated Hsp90ab1; reduced hepatic uptake is proposed but has not been demonstrated for this formulation. (**B**) Proposed intracellular availability of cyclophosphamide (CPA), its established cytotoxic actions, and the reported reduction in tumor-cell metabolic viability and extensive necrosis. Dashed arrows indicate proposed mechanisms; solid arrows depict CPA-associated effects. AG, arabinogalactan; ASGPR, asialoglycoprotein receptor [92,93,94].

Arabinogalactan from *L. sibirica* has also been investigated as a pharmaceutical carrier for reducing the effective dose and systemic toxicity of anticancer drugs (Figure 5). The arabinogalactan used by Zamay et al. was obtained from Siberian larch wood, consistent with wood being the only plant source from which this polysaccharide has been individually characterized in the compiled phytochemical literature [33,92]. However, the two arabinogalactan fractions reported previously, which accounted for 84.3% and 15.7% of the isolated polysaccharide fraction, were operational fractions obtained during isolation rather than independently characterized molecular species [33]. Zamay et al. did not establish whether their pharmaceutical-grade material corresponded to either fraction or represented a mixture [92]. Therefore, the relationship between the structural heterogeneity described in Ref. [33] and the drug-carrier properties evaluated by Zamay et al. remains unresolved.

Zamay et al. produced a cyclophosphamide-arabinogalactan mechanocomposite by processing the components for 12 h at a cyclophosphamide-to-arabinogalactan ratio of 1:10. The resulting material contained 9.1% cyclophosphamide and 90.9% arabinogalactan by total composite mass and was highly water-soluble, reaching approximately 250 g/L [92]. Spectroscopic analysis indicated that mechanochemical treatment did not chemically transform cyclophosphamide or generate covalent bonds between the drug and polysaccharide. Instead, cyclophosphamide became amorphous and molecularly dispersed within the arabinogalactan matrix, with the components stabilized principally through non-covalent hydrogen bonding [92,93]. This distinction is important because the preparation should be regarded as a mechanocomposite rather than a chemically defined cyclophosphamide-arabinogalactan conjugate. Although amorphization and molecular dispersion may improve dissolution and cellular availability, Zamay et al. did not report comparative release kinetics, colloidal stability, particle-size distribution or pharmacokinetic measurements [92]. Consequently, increased bioavailability remains a plausible explanation rather than a directly demonstrated mechanism.

To impart tumor selectivity, Zamay et al. functionalized the mechanocomposite with the DNA aptamer As42, which was previously selected against Ehrlich ascites carcinoma cells and was reported to recognize heat-shock-associated targets, including Hsp90ab1 [92,94]. The aptamer was added at 500 nM and associated with arabinogalactan through non-covalent interactions involving the nucleotide phosphate groups and polysaccharide hydroxyl groups [92]. This design was intended to address a central limitation of unmodified arabinogalactan: its exposed galactose-rich structures may bind asialoglycoprotein receptors that are highly expressed by hepatocytes, thereby promoting hepatic uptake rather than tumor-specific delivery [92,93,94,95,96,97]. Aptamer functionalization is therefore conceptually justified because it may alter the biological distribution of the carrier and provide an additional tumor-recognition element. Nevertheless, Zamay et al. did not quantify the aptamer density, binding stability in serum, receptor-specific cellular uptake or biodistribution of the cyclophosphamide-arabinogalactan-As42 complex [92]. The proposed targeting mechanism consequently remains insufficiently validated (Figure 5).

In Ehrlich ascites carcinoma cells, 5 µg/mL free cyclophosphamide caused approximately 70% cell death after 24 h, whereas the cyclophosphamide–arabinogalactan mechanocomposite caused complete cell death despite containing only 0.5 µg/mL cyclophosphamide. However, the difference between free cyclophosphamide and the mechanocomposite in the trypan-blue experiment was not statistically significant. In a separate MTT assay, 0.5 µg/mL free cyclophosphamide and 4.5 µg/mL arabinogalactan did not reduce cell viability, whereas 5 µg/mL free cyclophosphamide, the cyclophosphamide–arabinogalactan composite containing 0.5 µg/mL cyclophosphamide and its As42-functionalized form reduced metabolic viability by approximately 50% [92]. These results indicate that incorporation into the arabinogalactan matrix preserved cytotoxic activity at a tenfold lower cyclophosphamide concentration. Nevertheless, the MTT results did not demonstrate that As42 functionalization further increased cytotoxicity relative to the non-functionalized mechanocomposite. Moreover, MTT reduction measures cellular metabolic activity rather than cell death directly, while changes in intracellular sodium and potassium concentrations provide only indirect evidence of apoptosis or necrosis. The conclusion that the treatment induced both processes therefore requires confirmation using annexin V staining, caspase activation, membrane-integrity assays and cell-cycle analysis.

The in vivo experiment used ICR mice bearing intramuscularly transplanted Ehrlich carcinoma, with five animals assigned to each treatment group. Pure cyclophosphamide was administered at 5 µg/g, whereas the cyclophosphamide-arabinogalactan and cyclophosphamide–arabinogalactan–As42 preparations delivered only 0.5 µg/g cyclophosphamide within a total composite dose of 5 µg/g. Maximum survival increased from 20 days in untreated mice to 29 days with free cyclophosphamide, 59 days with the cyclophosphamide-arabinogalactan composite and 73 days with the As42-functionalized preparation. After Bonferroni correction, survival differed significantly from the untreated control for the cyclophosphamide–arabinogalactan and cyclophosphamide–arabinogalactan–As42 groups (*p* = 0.03198 and *p* = 0.03125, respectively), but not for free cyclophosphamide (*p* = 0.27207). Histological examination also revealed more extensive tumor necrosis and inflammatory infiltration after treatment with both arabinogalactan-containing formulations than after free cyclophosphamide treatment [92].

Despite the pronounced survival differences, several aspects restrict interpretation. First, Zamay et al. compared maximum survival rather than reporting median survival with hazard ratios and confidence intervals. Second, statistical comparisons were performed against the untreated control but not directly between cyclophosphamide-arabinogalactan and cyclophosphamide-arabinogalactan-As42. The increase from 59 to 73 days therefore does not establish a statistically significant contribution of the aptamer. Third, tumor volumes were not smaller in the composite-treated groups than in mice receiving free cyclophosphamide; the authors attributed this discrepancy to inflammatory infiltration, but inflammatory and viable tumor areas were not quantified histomorphometrically [92]. Finally, the use of only five animals per group limits statistical power, and the methods contain an internal inconsistency by stating that animals were divided into four groups while subsequently describing five groups.

The preliminary toxicity findings nevertheless support further evaluation. Free cyclophosphamide reduced the viability of primary mouse astrocytes and neurons, whereas arabinogalactan and both mechanocomposites produced no significant reduction in the MTT assay. In healthy mice, five injections of free cyclophosphamide reduced circulating neutrophil and monocyte proportions by approximately 27% and lowered lymphocyte abundance, while these changes were less pronounced after administration of the arabinogalactan-containing preparations. Free cyclophosphamide also increased alkaline phosphatase activity by more than twofold. By comparison, the biochemical parameters measured after treatment with the As42-functionalized complex remained close to control values. However, the non-functionalized cyclophosphamide-arabinogalactan composite increased serum cholesterol approximately 1.5-fold, which Zamay et al. attributed to hepatic uptake and metabolism of the carbohydrate carrier [92]. Because this explanation was not tested using tissue-distribution or metabolic-flux measurements, it should be considered a hypothesis rather than an established mechanism.

Earlier work by Zamay et al. showed that aptamer functionalization also increased the antitumor efficacy of a cisplatin-arabinogalactan preparation and permitted a 15-fold reduction in the cisplatin dose in Ehrlich carcinoma models [59]. Together, these studies suggest that aptamer-functionalized arabinogalactan may provide a versatile platform for delivering chemically distinct cytotoxic drugs. More broadly, aptamers offer high target affinity, relatively straightforward chemical modification and lower immunogenicity than many protein-based recognition ligands [95,96]. Nevertheless, these potential advantages do not establish translational suitability. The cyclophosphamide formulation still requires pharmacokinetic characterization, quantitative biodistribution, serum-stability testing, determination of aptamer binding affinity after composite formation, evaluation in orthotopic and molecularly defined tumor models and comparison with clinically established delivery systems. Thus, Zamay et al. provide promising proof-of-concept evidence for *L. sibirica* arabinogalactan as a cyclophosphamide carrier, but the available findings remain preclinical and do not yet demonstrate selective tumor delivery or clinical safety [92].

### 5.8. Hemocompatibility and Biomaterial Potential

**Figure 6:**
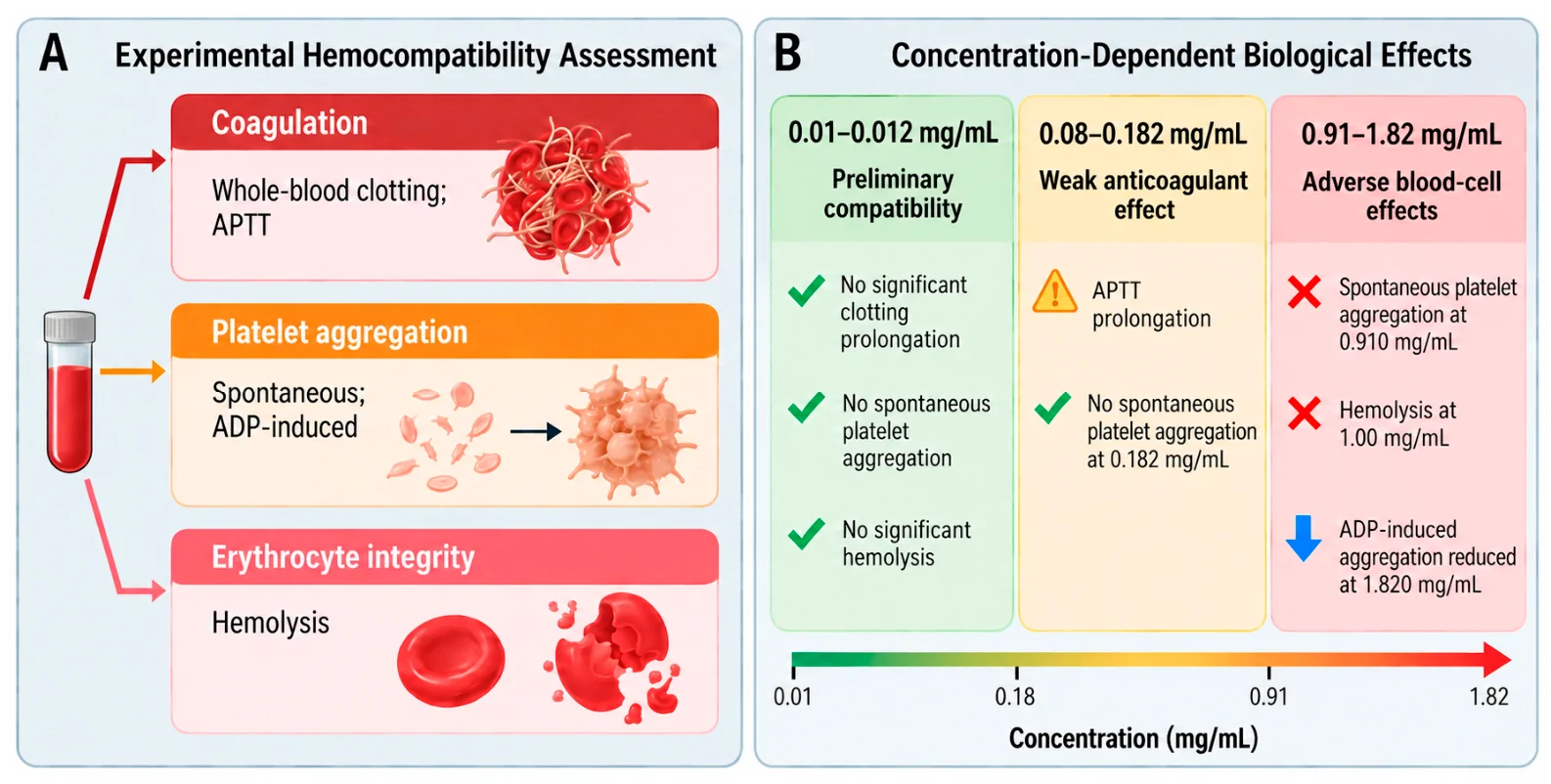
Concentration-dependent effects of sulfated *L. sibirica* organosolv lignins on blood components. (**A**) Assays used to assess whole-blood clotting and activated partial thromboplastin time (APTT), spontaneous and ADP-induced platelet aggregation, and erythrocyte integrity. (**B**) Summary of the reported responses: preliminary compatibility at low concentrations, weak anticoagulant activity at intermediate concentrations, and spontaneous platelet aggregation and hemolysis at higher concentrations. At 1.820 mg/mL, ADP-induced platelet aggregation was reduced. Green check marks indicate no significant adverse effect in the specified assay; the yellow symbol indicates APTT prolongation; red crosses indicate platelet activation or hemolysis. ADP, adenosine diphosphate [98].

The potential use of *L. sibirica*-derived lignin in blood-contacting biomaterials has been evaluated after chemical sulfation of organosolv lignin isolated from wood pulp (Figure 6). Drozd et al. investigated two water-soluble sulfated larch lignins, designated SLl-10 and SLl-11, together with five corresponding derivatives obtained from *Abies sibirica*. The two larch derivatives differed in sulfur content and molecular characteristics: SLl-10 contained 4.3% sulfur and had a weight-average molecular mass of 2960 Da, whereas SLl-11 contained 6.3% sulfur and had a molecular mass of 3497 Da [98]. Their dispersity values were 1.61 and 1.55, respectively. These relatively low molecular masses and complete water solubility support their potential incorporation into aqueous pharmaceutical formulations. However, sulfated lignins are chemically modified wood-derived polymers rather than native phytochemical constituents of *L. sibirica*. Their biological properties therefore reflect both the original lignin structure and the introduced sulfate groups.

Blood compatibility was assessed through complementary measurements of whole-blood and plasma coagulation, spontaneous and ADP-induced platelet aggregation, and erythrocyte membrane integrity. This multidimensional evaluation was necessary because the absence of an effect in one assay does not establish overall hemocompatibility. As emphasized by Gorbet et al., blood-contacting materials can activate coagulation factors, platelets, complement and inflammatory cells through partly independent processes [99]. At approximately 0.01–0.012 mg/mL, neither SLl-10 nor SLl-11 significantly prolonged whole-blood clotting, induced spontaneous platelet aggregation or increased erythrocyte hemolysis relative to the buffer control [98]. These findings indicate acceptable short-term compatibility at low concentrations and provide preliminary support for evaluating the two larch derivatives as components of drug-delivery systems.

Nevertheless, both larch lignins exhibited concentration-dependent effects on coagulation. The concentrations required to double activated partial thromboplastin time were 0.125 ± 0.026 mg/mL for SLl-10 and 0.080 ± 0.010 mg/mL for SLl-11. Corresponding antithrombin activities were only 0.634 ± 0.061 and 0.840 ± 0.106 U/mg, respectively, compared with 214.5 U/mg for the unfractionated heparin reference preparation [98]. Thus, the larch derivatives exhibited more than 250-fold lower antithrombin activity than heparin under the reported conditions. This weak activity contrasts with the stronger inhibition of thrombin and factor Xa reported for some structurally optimized sulfated low-molecular-weight lignins [100]. Sulfation alone is therefore insufficient to confer potent anticoagulant activity; molecular mass, sulfate distribution, aromatic structure and three-dimensional conformation may also determine interactions with coagulation proteases.

The platelet results further demonstrate that compatibility was restricted to a defined concentration range. At 0.182 mg/mL, SLl-10 and SLl-11 did not provoke spontaneous platelet aggregation. However, increasing the concentration to 0.910 mg/mL induced spontaneous aggregation among the tested sulfated lignins, with reported responses ranging from 45.2 ± 7.3% to 68.0 ± 4.5% [98]. At 1.820 mg/mL, both larch derivatives inhibited ADP-induced aggregation, but SLl-10 was more active, reducing aggregation to 29.17 ± 4.59%, whereas SLl-11 reduced it to approximately 50%, compared with 72.11 ± 2.09% in the ADP control. The higher sulfur content of SLl-11 therefore did not produce stronger antiaggregant activity. Drozd et al. suggested that the higher sulfation temperature and longer reaction time used to produce SLl-11 may have reduced its inhibitory activity [98]. However, this explanation remains provisional because only two larch preparations were compared, and their sulfate substitution patterns were not characterized in sufficient detail.

Erythrocyte compatibility was similarly concentration-dependent. At 0.01 mg/mL, the optical densities measured after incubation of erythrocytes with SLl-10 and SLl-11 were 0.0510 ± 0.0054 and 0.0543 ± 0.0100, respectively, and did not differ significantly from the buffer control value of 0.0473 ± 0.0091. In contrast, increasing the concentration to 1.00 mg/mL raised the corresponding values to 0.9414 ± 0.0314 and 1.0190 ± 0.0326, compared with 0.0718 ± 0.0112 in the negative control [98]. These increases indicate substantial disruption of erythrocyte membranes at the higher concentration. Accordingly, describing the larch lignins as hemocompatible without specifying the tested concentration would be misleading. The results support compatibility near 0.01 mg/mL, but they also identify clear platelet-activating and hemolytic effects at concentrations approaching 1 mg/mL.

The findings have different implications for drug carriers and thromboresistant biomaterials. The low-concentration results justify further evaluation of SLl-10 and SLl-11 as structural components of drug-delivery systems, particularly because lignin-based materials can be formulated as nanoparticles, hydrogels, membranes and drug-release matrices [101]. In contrast, the evidence does not establish the larch derivatives as effective anticoagulant coatings. The derivative that combined prolonged coagulation with reduced ADP-induced platelet aggregation over a broader concentration range was SLf-6 from *A. sibirica*, not either *L. sibirica* preparation [98]. Therefore, the proposed use of sulfated lignin for thromboresistant surfaces is supported more strongly by the fir-derived material, whereas the larch derivatives remain preliminary candidates requiring further structural optimization.

Overall, Drozd et al. demonstrated that sulfated *L. sibirica* lignins can interact with several components of human hemostasis, but their effects were strongly dependent on concentration and preparation conditions [98]. The study was limited to short-term in vitro assays and included only 5–17 independent measurements, depending on the experiment. It did not evaluate complement activation, leukocyte responses, endothelial compatibility, protein adsorption, repeated exposure, degradation products or thrombosis in vivo. Furthermore, the platelet and hemolysis assays revealed adverse effects at higher concentrations, preventing extrapolation of low-concentration compatibility to implanted devices or systemic administration. Consequently, future studies should establish concentration–response relationships under physiologically relevant flow conditions, characterize sulfate substitution patterns, compare multiple *L. sibirica* lignin preparations and evaluate both local and systemic compatibility in vivo before these materials are considered for drug-delivery systems or blood-contacting materials.

## 6. Research Gaps and Future Directions

Despite the substantial number of compounds reported from *L. sibirica*, the available phytochemical evidence remains uneven across plant tissues, compound classes and analytical periods. Early investigations concentrated on the isolation of selected flavonoids from bark, diterpenoids from oleoresin and flavonoid glycosides from needles [25,26,27,28,29,30,31]. These studies established the principal chemical classes of the species, but they were usually designed to isolate individual compounds rather than characterize complete metabolite profiles. Bark, needles, oleoresin, heartwood resin and woody greens consequently account for most of the reported chemical diversity, whereas roots, cambial tissues, buds, flowers and reproductive structures remain comparatively underinvestigated [25,26,27,28,29,30,31,32,33,34,35,36,37,38,39,40,41,42,43,44,45,46,47,48,49,50,51,52,53,54,55,56]. The identification of prostaglandins in the cambial zone and buds and volatile benzenoid derivatives in female flowers illustrates that less frequently examined tissues may contain chemically distinctive constituents [34,35]. Future sampling should therefore extend beyond the tissues already recognized as convenient sources of phenolics, terpenoids and arabinogalactan.

Analytical development represents a second major priority. Many early studies relied on paper chromatography, column chromatography, UV and IR spectroscopy or gas–liquid chromatography, often without the accurate mass measurements, fragmentation data or authentic standards expected for contemporary structural annotation [25,26,27,28,29,30,31,32]. Although later investigations introduced NMR, GC-MS, GC-FID and more selective chromatographic approaches [37,38,39,40,41,42,43,44,45,46,47,48,49,50,51,52,53,54,55], these studies generally remained targeted toward predefined fractions or compound classes. For example, recent work has characterized knotwood lignans by supercritical fluid chromatography, sterols in cultured cells and volatile terpenes in woody tissues, but these analyses do not provide a comprehensive representation of the total metabolome [52,53,55]. Untargeted LC-HRMS and GC-HRMS profiling should therefore be applied across standardized tissue sets, followed by MS/MS-based molecular networking and confirmation of high-priority annotations using authentic standards and NMR. Importantly, tentative library matches should be distinguished from compounds confirmed by retention time, fragmentation and reference materials.

Quantitative characterization is even less developed than qualitative identification. Several investigations reported only the presence, trace occurrence or predominance of a compound, whereas numerical measurements were expressed relative to different matrices, including dry tissue, essential oil, total extract, resinous compounds, isolated fractions, extraction yield or product purity [25,26,27,28,29,30,31,32,33,34,35,36,37,38,39,40,41,42,43,44,45,46,47,48,49,50,51,52,53,54,55,56]. These values cannot be combined directly because they describe different analytical denominators. Extraction yield and product purity, for example, do not represent the native concentration of a compound in the original tissue. Future studies should report concentrations on a common dry-weight basis wherever possible and provide extraction recovery, internal standards, calibration ranges, limits of detection, limits of quantification and matrix-effect assessments. Validated LC-MS/MS and GC-MS methods are particularly needed for simultaneous quantification of taxifolin, phenolic acids, lignans, resin acids, sterols and major volatile terpenoids. Certified or well-characterized reference materials would further improve comparisons among laboratories and production batches.

Biological and environmental variation also requires more systematic investigation. Seasonal differences have been examined in oleoresin, crown material and needles [27,44,46,48], while more recent studies have evaluated geographical variation in volatile compounds, responses to industrial pollution, lipid remodeling and latitudinal variation in constitutive chemical defenses [45,49,50,54,56]. Collectively, these studies indicate that phytochemical composition is not a fixed species-level property. However, they differ in sampling location, tissue age, harvest period, extraction procedure and analytical platform, making it difficult to separate the effects of genotype, climate, developmental stage and environmental exposure. Future studies should use factorial sampling designs that include multiple populations, seasons, developmental stages and biological replicates analyzed using the same validated workflow. Voucher specimens, precise geographic coordinates, environmental metadata and tissue-development criteria should also be reported to improve reproducibility.

Cell cultures represent another potentially valuable but insufficiently characterized source of *L. sibirica* metabolites. Semenova et al. demonstrated differences in sterol composition between embryogenic and nonembryogenic cell lines, indicating that developmental state can alter phytosterol metabolism [52]. Nevertheless, evidence from a limited number of cultured lines cannot establish whether these profiles are stable across genotypes, passages or culture conditions. Comparative studies should evaluate donor genotype, culture age, growth regulators, nutrient composition and elicitor exposure. Parallel analysis of cultured cells and the corresponding intact tissues would clarify which metabolic pathways are retained, suppressed or induced in vitro. Such work could determine whether cell cultures offer a reproducible platform for producing selected sterols, phenolics or terpenoids rather than merely serving as experimental models of plant development.

Extraction and processing studies likewise remain concentrated on conventional procedures. The reviewed literature predominantly used solvent maceration, sequential liquid–liquid partitioning, Soxhlet extraction, steam distillation, precipitation and column chromatography [25,26,27,28,29,30,31,32,33,34,35,36,37,38,39,40,41,42,43,44,45,46,47,48,49,50,51,52,53,54,55,56]. Levdansky et al. optimized aqueous ethanol extraction for the simultaneous recovery of dihydroquercetin and arabinogalactan from wood, demonstrating that 15% ethanol at 90–100 °C for 2 h provided 1.8% and 18.0% yields, respectively [51]. However, comparable optimization has not been performed for most other tissues or compound classes. Moreover, supercritical fluid chromatography was used for downstream analytical and preparative separation of knotwood lignans rather than primary extraction of the plant material [53]. Claims concerning the advantages of supercritical CO_2_, microwave-assisted, ultrasound-assisted or enzyme-assisted extraction for *L. sibirica* therefore remain largely hypothetical. Direct method comparisons should evaluate recovery, selectivity, degradation, solvent and energy consumption, reproducibility and scalability using the same starting material.

The relationship between chemical composition and biological activity also remains incompletely defined. Studies of bark antioxidants and neuritogenic phenolics demonstrate that isolated constituents can possess measurable biological activity [39,41], but most extracts contain multiple compounds whose individual contributions and interactions have not been resolved. Bioactivity-guided fractionation should be combined with quantitative chemical profiling to identify which constituents account for observed effects. Structure–activity comparisons should include closely related compounds, such as taxifolin and aromadendrin, phenolic acids with different hydroxylation patterns, structurally related lignans and individual abietane- and pimarane-type resin acids. However, correlations between metabolite abundance and activity should not be interpreted as evidence of causation without testing purified compounds, defined mixtures and appropriate concentration-response relationships.

Finally, pharmacological translation is limited by the predominance of chemical assays, cell models and small preclinical studies. Systematic information on absorption, metabolism, tissue distribution, bioavailability, repeated-dose toxicity and interactions with conventional medicines remains scarce. This limitation is especially important for modified arabinogalactans, drug–arabinogalactan formulations and sulfated lignins because chemical modification may alter distribution and toxicity relative to the native polymers. Future studies should first establish chemically standardized test materials and then apply dose–response experiments, pharmacokinetic analysis, relevant disease models and adequately powered toxicity studies. Controlled clinical investigation would be justified only after the identity, composition, dose and safety of the tested preparation have been defined. Evidence obtained from other *Larix* species or from isolated commercial compounds should not be attributed directly to *L. sibirica* unless the botanical source and chemical equivalence have been confirmed.

The literature search was conducted between 10 April and 15 July 2026 without restrictions on publication year or language. Although relevant studies published in English, Russian and Chinese were identified, regional publications not indexed in the searched databases may have been missed. Grey literature was not searched systematically. The review may also be affected by publication bias if studies reporting positive findings were more likely to be published than studies reporting null results. These limitations should be considered when interpreting the completeness of the evidence and the strength of the conclusions.

Future research should shift from the continued reporting of isolated compounds toward integrated, quantitative and reproducible investigation of *L. sibirica*. The most immediate priorities are comprehensive metabolomic coverage of underexamined tissues, validated multi-analyte quantification, standardized sampling and extraction, controlled assessment of environmental and developmental variation, and direct linkage of defined chemical profiles to biological effects. Addressing these priorities would provide the evidence required to distinguish promising phytochemical leads from findings that remain descriptive, method-dependent or insufficiently validated.

## 7. Conclusions

*L. sibirica* contains a chemically diverse but unevenly characterized phytochemical repertoire. The compiled evidence comprises 233 unique compounds distributed among 16 phytochemical groups, with phenolics, terpenoids, lipids, lignans and arabinogalactan representing the principal constituents. Their distribution is strongly tissue-dependent: bark is distinguished by phenolic acids, flavonoids and catechins; oleoresin and resinous tissues by volatile terpenoids, diterpenes and resin acids; needles by lipids, sterols and wax-related compounds; knotwood by lignans; and wood by arabinogalactan. However, this apparent specialization also reflects unequal research intensity, and the composition of roots, reproductive structures, cambial tissues and other less frequently sampled sources remains poorly resolved.

No single extraction procedure is suitable for this chemical diversity. Conventional solvent extraction, partitioning, distillation and chromatography remain the dominant approaches, while aqueous ethanol offers a practical strategy for recovering arabinogalactan and taxifolin from wood. Nevertheless, differences in tissue preparation, solvent composition, extraction conditions and measurement basis prevent rigorous comparison of yields across studies. Claims concerning emerging extraction technologies remain premature because direct comparisons under standardized conditions are largely unavailable.

The pharmacological evidence identifies credible biological activity but varies considerably in strength. Radical-scavenging effects of phenolics and lignans are supported mainly by chemical assays, while antiviral, antibacterial, neuritogenic, anti-inflammatory and anti-allergic activities depend predominantly on cellular or limited animal models. Arabinogalactan has shown immunomodulatory, intestinal barrier and drug-carrier potential, whereas sulfated lignins have demonstrated concentration-dependent interactions with coagulation, platelets and erythrocytes. These findings support continued preclinical development but do not establish clinical efficacy, universal hemocompatibility or safety. Progress will require chemically standardized preparations, validated quantitative profiling, adequately powered dose–response studies, pharmacokinetic characterization and systematic toxicological evaluation. Until these requirements are met, *L. sibirica* should be regarded as a promising source of phytochemicals and functional biopolymers rather than an established source of clinically validated therapeutics.

## Figures and Tables

**Table 1:** Chronological summary of phytochemical studies on *L. sibirica*.

Year	Plant Source(s)	Sample Preparation	Analytical Method(s)	Research Focus	Ref.
1967	Bark	Ether extraction after petroleum ether/chloroform pretreatment; silica gel purification; recrystallization from 70% methanol	PC; CC; UV	Isolation and identification of flavonols	[25]
1967	Oleoresin (neutral fraction)	Isolation of diterpene diol; oxidation, hydrogenation and reduction	IR; NMR	Structural elucidation of a diterpene diol	[26]
1968	Oleoresin (resin)	Resin dissolved in acetone	GC-FID	Comparative analysis of monoterpene composition and seasonal variation	[27]
1970	Bark	Acetone extraction; bicarbonate treatment; ethyl acetate partitioning; preparative purification	PC; UV	Isolation and identification of flavonol glycosides and dihydroflavonols	[28]
1970	Bark	Methanol extraction after petroleum ether/benzene pretreatment; tannin precipitation; purification	PC; IR; UV	Isolation and identification of catechins	[29]
1974	Needles	Methanol extraction; solvent partitioning; polyamide purification	UV; IR; ^1^H NMR; GLC; PC	Isolation and structural identification of new flavonol glycosides	[30]
1975	Oleoresin	Fractionation into terpene fractions	GLC; IR; NMR	Characterization of monoterpenoids and sesquiterpenoids	[31]
1982	Woody verdure	Steam distillation; essential oil fractionation	GLC; IR; TLC	Characterization of essential oil composition and isolation of sesquiterpene hydrocarbons	[32]
1984	Wood (extractive-free sawdust)	Cold-water extraction; ethanol precipitation; Sephadex G-75 gel filtration; partial acid hydrolysis; methylation and enzymatic oxidation of purified arabinogalactan	^13^C NMR; GLC; GC-MS; PC	Structural characterization of wood arabinogalactan	[33]
1985	Female flowers	Dynamic headspace collection on TENAX GC; n-pentane elution	GC-MS	Characterization of volatile odoriferous compounds	[34]
1988	Cambial zone and buds	Ethanol extraction; TLC purification; derivatization of prostaglandins (methylation and trimethylsilylation)	TLC; UV; IR; GC; GC-MS; GC-SIMS; HPLC; ^1^H NMR	Identification and seasonal variation in prostaglandins	[35]
1994	Needles and shoots (woody verdure)	Total lipid extraction; silica gel CC isolation of glycolipids; two-dimensional TLC purification; alkaline and acid hydrolysis	TLC; GLC; IR; PC	Characterization of glycolipid composition, fatty acid profiles, and seasonal dynamics	[36]
2003	Bark	Ethyl acetate extraction; flash chromatography; fractionation and rechromatography of phenolic fractions; acetylation of isolated compounds	TLC; HPLC; UV; IR; ^1^H NMR; ^13^C NMR; HMQC; HMBC; COSY; NOESY; JMOD	Comprehensive characterization of bark phenolic compounds (phenolic acids, flavonoids, biflavonoids, proanthocyanidins, and condensed tannins)	[37]
2006	Bark	Bark extract fractionation by chromatography; isolation of spiroflavonoid-containing fractions; acetylation of the isolated compound	TLC; IR; ^1^H NMR; ^13^C NMR; JMOD	Isolation and structural elucidation of a new spiroflavonoid (triflarixinol)	[38]
2008	Outer bark	Isolation of phenolcarboxylic acids from outer bark; purification (>90% by HPLC)	HPLC; manometric oxygen uptake assay	Quantitative evaluation of antioxidant activity of bark phenolcarboxylic acids	[39]
2011	Heartwood resin (ethyl acetate extract)	Ethyl acetate extraction of heartwood chips; fractionation into acidic and neutral fractions; solvent fractionation; derivatization (trimethylsilylation/methylation)	HPLC; GC; GC–MS; TLC	Comprehensive characterization of heartwood resin constituents and identification of isocembrol	[40]
2014	Knot wood	70% aqueous isopropanol extraction; silica gel flash column chromatography; crystallization of purified phenolic compounds	HPLC; ^1^H NMR; HRMS; Polarimetry	Isolation of phenolic compounds and evaluation of their neuroactive properties	[41]
2014	Branch bark	Bark manually separated from branches, milled (1–2 mm), Soxhlet extraction with isopropanol, petroleum ether partitioning, silica gel column chromatography, AgNO_3_-impregnated silica gel fractionation	GC–MS; ^1^H NMR; ^13^C NMR; TLC; IR spectroscopy	Characterization of neutral hydrocarbons and terpene composition of branch bark extractives	[42]
2015	Wood (xylem lignin)	Dioxane lignin isolated from resin-free wood using aqueous dioxane/HCl, purified by reprecipitation and lyophilization	2D NMR (HSQC, HMBC, COSY); ^13^C NMR	Structural characterization of lignin and identification of lignin interunit linkages and aromatic units	[43]
2015	Crown (needles, twig bark, woody part of twigs, fallen needles)	Sequential isopropanol extraction followed by liquid–liquid fractionation with petroleum ether, diethyl ether and ethyl acetate; acid–base fractionation of lipophilic and phenolic fractions	IR spectroscopy; TLC	Comparative group composition of lipophilic and phenolic extractives from different crown tissues collected in summer and autumn	[44]
2018	Needles	Needle sampling from different urban functional zones; needle discs prepared for surface analysis	SEM–EDS; pigment and biochemical assays	Physiological and biochemical adaptation of needles to urban pollution through changes in particle deposition, pigments and antioxidant metabolites	[45]
2019	Needles (green and yellow)	Sequential solvent extraction (propan-2-ol, petroleum ether, diethyl ether, ethyl acetate); fractionation into resinous, neutral and acidic fractions; wax isolation; methylation of acids	UV; TLC; FTIR; ^1^H/^13^C NMR; GC–MS	Seasonal variation in extractive composition, waxes, free and bound fatty/resin acids in needles	[46]
2019	Needles	Propan-2-ol extraction; petroleum ether fractionation; silica gel column purification; alkaline hydrolysis of ester fraction; acetylation	TLC; FTIR; ^1^H/^13^C NMR; GC–MS	Isolation and identification of triterpene alcohols and phytosterols from needle esters	[47]
2020	Needles (green and yellow)	Sequential solvent extraction; fractionation into neutral, acidic and wax fractions; acid methylation	TLC; GLC; UV; FTIR; ^1^H/^13^C NMR; GC–MS	Seasonal variation in needle extractives, waxes, and free/bound fatty and resin acids	[48]
2022	Twigs	Freshly collected twigs; steam distillation under atmospheric pressure; 50 essential oil samples collected from natural populations	GC–MS; GC-FID; GC × GC (chiral); ^1^H/^13^C NMR (reference compounds)	Comprehensive characterization, geographical variation, authentication, and quality control of Siberian conifer essential oils	[49]
2022	Needles	Needles collected from 80-year-old trees across pollution gradients; dried and ground for elemental analysis; fresh needles frozen in liquid nitrogen for antioxidant assays	ICP-AES (SPECTRO ARCOS); spectrophotometry (H_2_O_2_, ascorbate, glutathione, phenolics, proline); Folin–Ciocalteu assay; AlCl_3_ flavonoid assay; statistical cluster analysis	Evaluation of low-molecular-weight antioxidant responses (proline, phenolics, ascorbate, glutathione) of *L. sibirica* under industrial (aluminum smelter) pollution and oxidative stress	[50]
2023	Wood	Wood milled to 1–3 mm particles; extraction with 5–25% aqueous ethanol; mechanical activation; isolation of dihydroquercetin and arabinogalactan	FTIR; ^13^C NMR; TLC; UV–Vis spectrophotometry	Optimization of simultaneous extraction and structural characterization of dihydroquercetin and arabinogalactan from larch wood	[51]
2023	Embryogenic and nonembryogenic cell lines	In vitro cultured callus tissues; lipid extraction with chloroform:methanol; TLC isolation of sterol fractions; trimethylsilylation prior to GC analysis	TLC; GC–MS; mass spectral identification (NIST/Wiley libraries); statistical analysis	Comparative profiling of free sterols and sterol esters associated with somatic embryogenesis and embryogenic potential in *L. sibirica* cell cultures	[52]
2023	Knotwood	Soxhlet extraction after hexane de-resination; acetone extraction; freeze-drying of extracts	SFC; HRMS (APCI-QTOF-MS); NMR (^1^H, HSQC, HMBC); HPLC-UV	Analytical and semi-preparative separation, purification, and identification of lignans from softwood knotwood	[53]
2025	Needles	Liquid nitrogen fixation; Folch lipid extraction (chloroform:methanol); TLC fractionation of lipid classes	TLC; ICP-AES; spectrophotometry; densitometry	Lipid profiling and characterization of membrane lipid adaptations under chronic industrial pollution	[54]
2025	Woody greens	Woody greens ground (1–2 mm); Soxhlet extraction with petroleum ether, diethyl ether, ethyl acetate and isopropanol; acid–base fractionation; acetylation of alcohol fractions	GC–MS; TLC; CC; ^1^H/^13^C NMR	Comparative characterization of neutral alcohols and diterpenoids in woody greens of Siberian and European larch	[55]
2025	Spring needles	Air-dried needles; Soxhlet extraction with isopropanol; petroleum ether extraction; derivatization; alumina fractionation	GC–MS	Geographic variation in constitutive chemical-defense compounds along a latitudinal gradient	[56]

CC, column chromatography; COSY, correlation spectroscopy; FTIR, Fourier-transform infrared spectroscopy; FT-NIR, Fourier-transform near-infrared spectroscopy; FT-Raman, Fourier-transform Raman spectroscopy; GC, gas chromatography; GC-FID, gas chromatography with flame ionization detection; GC-MS, gas chromatography–mass spectrometry; GC-SIMS, gas chromatography–selected ion monitoring mass spectrometry; GLC, gas–liquid chromatography; HMBC, heteronuclear multiple bond correlation; HMQC, heteronuclear multiple quantum coherence; HPLC, high-performance liquid chromatography; HRMS, high-resolution mass spectrometry; HR-LC/Q-TOF/MS, high-resolution liquid chromatography/quadrupole time-of-flight mass spectrometry; ICP-AES, inductively coupled plasma atomic emission spectroscopy; IR, infrared spectroscopy; JMOD, J-modulated ^13^C nuclear magnetic resonance experiment; NMR, nuclear magnetic resonance spectroscopy; ^1^H NMR, proton nuclear magnetic resonance spectroscopy; ^13^C NMR, carbon-13 nuclear magnetic resonance spectroscopy; PC, paper chromatography; SFC, supercritical fluid chromatography; SEM-EDS, scanning electron microscopy coupled with energy-dispersive X-ray spectroscopy; TLC, thin-layer chromatography; UV, ultraviolet spectroscopy.

**Table 2:** Major classes of compounds reported from *Larix sibirica*.

Compound Class	Unique Compounds, n	Main Plant Sources	Representative Reported Result
Terpenoids and diterpenoids	131	Oleoresin, branch bark, needles, woody greens, heartwood resin	3-Carene: 49.0% of oleoresin (mean) [27]
Flavonoids	33	Bark, needles, knotwood, heartwood resin	Taxifolin: 12.0% of total heartwood resinous compounds [40]
Fatty acids and other lipids	27	Needles, cambial zone, buds	Myristic acid: 0.26–6.60% in needles, as reported [46]
Triterpenoids and phytosterols	13	Needles, woody greens, cultured cells	Stigmasterol: 21.8–248.2 μg/g dry weight in cultured cells [52]
Phenolic acids and other polyphenols	12	Bark, needles, heartwood resin	*p*-Hydroxybenzoic acid: trace–0.18% in needles [46]
Lignans and oligolignans	7	Knotwood, heartwood resin	(−)-Secoisolariciresinol: 23% isolation yield from knotwood; 91% product purity [53]
Long-chain alcohols, ketones and volatile benzenoids	7	Needle wax, woody greens, female flowers	Nonacosan-10-ol: 95.5% of the needle wax fraction [46]
Polysaccharides	2	Wood	Arabinogalactan A: 84.3% of the isolated polysaccharide fraction [33]

## Data Availability

No new data were created or analyzed in this study. Data sharing is not applicable to this article.

## Supplementary Materials

The following supporting information can be downloaded at: https://www.mdpi.com/article/10.3390/molecules31203690/s1. Table S1: Record-level database of phytochemical constituents reported in *L. sibirica*, including plant sources, references, and quantitative data; Table S2: Phenolic acids, flavonoids, catechins, proanthocyanidins, tannins, lignans, and oligolignans reported in *L. sibirica*, including plant sources, references, and quantitative data; Table S3: Terpenoids, resin acids, and related compounds reported in *L. sibirica*, including plant sources, references, and quantitative data; Table S4: Fatty acids, long-chain alcohols, ketones, and wax constituents reported in *L. sibirica*, including plant sources, references, and quantitative data.

## Abbreviations

The following abbreviations are used in this manuscript:

APCI-QTOF-MS	Atmospheric pressure chemical ionization quadrupole time-of-flight mass spectrometry
CC	Column chromatography
COSY	Correlation spectroscopy
EDS	Energy-dispersive X-ray spectroscopy
FTIR	Fourier-transform infrared spectroscopy
FT-NIR	Fourier-transform near-infrared spectroscopy
FT-Raman	Fourier-transform Raman spectroscopy
GC	Gas chromatography
GC × GC	Comprehensive two-dimensional gas chromatography
GC-FID	Gas chromatography with flame ionization detection
GC-MS	Gas chromatography-mass spectrometry
GC-SIMS	Gas chromatography-selected ion monitoring mass spectrometry
GLC	Gas–liquid chromatography
HMBC	Heteronuclear multiple-bond correlation
HMQC	Heteronuclear multiple-quantum coherence
HPLC	High-performance liquid chromatography
HPLC-UV	High-performance liquid chromatography with ultraviolet detection
HRMS	High-resolution mass spectrometry
HR-LC/Q-TOF/MS	High-resolution liquid chromatography coupled with quadrupole time-of-flight mass spectrometry
HSQC	Heteronuclear single-quantum coherence
ICP-AES	Inductively coupled plasma atomic emission spectroscopy
IR	Infrared spectroscopy
JMOD	J-modulated carbon-13 nuclear magnetic resonance experiment
LC-MS/MS	Liquid chromatography-tandem mass spectrometry
NIST	National Institute of Standards and Technology
NMR	Nuclear magnetic resonance spectroscopy
^1^H NMR	Proton nuclear magnetic resonance spectroscopy
^13^C NMR	Carbon-13 nuclear magnetic resonance spectroscopy
NOESY	Nuclear Overhauser effect spectroscopy
PC	Paper chromatography
PRISMA-S	Preferred Reporting Items for Systematic Reviews and Meta-Analyses literature search extension
Q-TOF	Quadrupole time-of-flight
SEM	Scanning electron microscopy
SEM-EDS	Scanning electron microscopy coupled with energy-dispersive X-ray spectroscopy
SFC	Supercritical fluid chromatography
TLC	Thin-layer chromatography
UV	Ultraviolet spectroscopy
PGA_2_	Prostaglandin A_2_
PGB_2_	Prostaglandin B_2_
PGE_1_	Prostaglandin E_1_
PGE_2_	Prostaglandin E_2_
PGF_1_α	Prostaglandin F_1_α
PGF_2_α	Prostaglandin F_2_α
